# Emerging biomarkers for Parkinson's disease in biological fluids

**DOI:** 10.3389/fnagi.2026.1835751

**Published:** 2026-07-13

**Authors:** Petra Bago Rožanković, Goran Šimić

**Affiliations:** 1 Department of Neurology, University Hospital Dubrava, Avenija Gojka Šuška, Zagreb, Croatia; 2 School of Medicine, Catholic University of Croatia, Ilica, Zagreb, Croatia; 3 Department of Neuroscience, Croatian Institute for Brain Research, University of Zagreb School of Medicine, Šalata, Zagreb, Croatia

**Keywords:** Alzheimer's disease biomarkers, extracellular vesicles, neuroinflammation, Parkinson's disease, seed amplification assays (SAA), α-synuclein

## Abstract

Early and accurate diagnosis of Parkinson's disease (PD) remains a challenge, hindering the efficient recruitment of patients into clinical trials aimed at disease modification. This underscores the urgent need for validated and clinically applicable biomarkers. Research continues to expand our knowledge of fluid biomarkers for detecting and monitoring PD progression. Cerebrospinal fluid α-synuclein (α-syn) seed amplification assays (SAA) have emerged as highly sensitive and specific biomarkers for the diagnosis of PD. The development of less invasive procedures using biological fluids such as serum or saliva would be more practical for routine clinical use. Recent data demonstrate the presence of pathogenic α-synuclein in the serum of PD patients compared with healthy controls, detected using real-time quaking-induced conversion (RT-QuIC) assays. Elevated ratios of pS129-α-syn and/or oligomeric α-syn to total α-syn have also been reported in PD. Future research should clarify differences in protein aggregate formation across various synucleinopathies. Promising advances toward a clinically useful blood-based diagnostic test for PD include the quantification of proteins released from neural-derived extracellular vesicles (NDEVs), such as oligomeric and phosphorylated α-syn, tau, and disease-associated microRNAs. Given the role of neuroinflammation in PD pathogenesis, inflammatory biomarkers, including interleukin (IL)-6, IL-10, tumor necrosis factor-α(TNF-α), glial fibrillary acidic protein (GFAP), chitinase-3-like protein 1 (YKL-40), and monocyte chemoattractant protein-1 (MCP-1), are under active investigation. Furthermore, fluid biomarkers associated with Alzheimer's disease pathology are being explored for their potential to predict motor and cognitive decline in PD and related synucleinopathies. Salivary EVs hold promise as a non-invasive source of PD biomarkers; however, robust validation in large, well-characterized cohorts is essential to improve the diagnostic and prognostic accuracy of PD.

## 1 Introduction

Parkinson's disease (PD) is the second most common neurodegenerative disorder, characterized by core motor and non-motor symptoms, as defined by current diagnostic criteria (Lee and Gilbert, 2016; Postuma et al., 2015). However, these clinical features overlap with other atypical or secondary parkinsonian syndromes, leading to misdiagnosis in up to 20% of cases (Rizzo et al., 2016). This emphasizes the need for reliable, clinically applicable biomarkers of PD to optimize diagnosis and treatment development.

A biomarker is defined as a characteristic that is objectively measured and evaluated as an indicator of normal biological processes, pathogenic processes, or pharmacologic responses to a therapeutic intervention. Biomarkers are used for diagnosis, evaluation of disease severity and prognosis, and prediction of responsiveness to therapeutic intervention. Various factors could be considered biomarkers, including clinical signs, biochemical specimens, imaging findings, and genetic data (Biomarkers Definitions Working Group, 2001).

This review provides a distinct perspective by integrating evidence from α-synuclein (α-syn) seed amplification assays (SAA) with extracellular vesicle (EV) cargo across multiple biofluids, with a special focus on saliva-derived vesicles. It discusses methodological pitfalls, cross-cohort reproducibility, and the translational path toward clinically useful multi-marker panels. We explicitly call out gaps in the literature: limited standardization of saliva EV isolation/analysis, insufficient longitudinal data linking salivary EV cargo to clinical trajectories, and the need for head-to-head comparisons of EV sources from saliva, plasma, and neuron-derived EVs (NDEVs) within the same cohorts. Here, by standardization, we mean the adoption of widely accepted guidelines, procedures, and consensus protocols such as those recommended for Minimal Information for Studies of Extracellular Vesicles (MISEV, Welsh et al., 2024; for review see Guzowska et al., 2026), which would include cargo normalization (e.g., relative to vesicle number, total protein, or neuron-enriched markers) and commercially available kits and assay platforms that are scalable and cost-effective for clinical use, to achieve consistent and comparable results across different laboratories.

Biomarkers with the potential to detect the early stages of PD or differentiate it from other parkinsonian disorders could contribute to better patient recruitment in clinical trials targeting disease modification. Furthermore, reliable biomarkers could contribute to more accurate disease prognosis and assessment of treatment response in clinical trials (Delenclos et al., 2016).

In recent years, research on biomarkers in biological fluids has expanded, providing new data for the diagnosis of PD. Cerebrospinal fluid (CSF) α-syn SAA have been validated as highly sensitive and specific biomarkers for PD diagnosis (Koga et al., 2021; Kang et al., 2019; Russo et al., 2021; Bellomo et al., 2022; Siderowf et al., 2023; Grossauer et al., 2023; Poggiolini et al., 2022). In distinguishing PD from other disorders, serum α-syn levels, including total, phosphorylated, and oligomeric variants, have been measured using real-time quaking-induced conversion (RT-QuIC) assays (Rossi et al., 2020; Okuzumi et al., 2023; Pedersen et al., 2025; Stuendl et al., 2021; Parnetti et al., 2019; Zubelzu et al., 2022). However, further reliable quantifications and identification of specific pathological patterns of α-syn aggregates are needed to distinguish different synucleinopathies (Ganguly et al., 2021). Promising advances include the quantification of α-syn variants, tau protein, and disease-associated microRNAs from neuron-derived extracellular vesicles (NDEVs) (Jiang et al., 2020; Zhao and Yang, 2021; Xylaki et al., 2023; Ohmichi et al., 2019; Si et al., 2019; Niu et al., 2020; Ma et al., 2025). Additionally, inflammatory biomarkers and those associated with Alzheimer's disease (AD) have been explored for their potential in diagnosing PD (Coughlin and Irwin, 2023; Zimmerman and Brockman, 2022; Lin et al., 2025).

This review summarizes current knowledge of specific biomarkers for PD in biological fluids, with a special emphasis on their roles in early diagnosis and disease progression.

## 2 Alpha-synuclein biomarkers

Alpha-synuclein (α-syn) misfolding plays a central role in the pathogenesis of PD. Post-translational modifications, such as phosphorylation of α-syn, and the facilitation of misfolding of α-syn by various processes, including genetic mutations, oxidative stress, and impaired function of the ubiquitin-proteasome system, result in the formation of oligomeric forms, insoluble fibrils, and ultimately Lewy bodies, a pathological hallmark of PD (Li X. Y. et al., 2021; Poewe et al., 2017; Schmid et al., 2013). Therefore, levels of total, phosphorylated, and oligomeric α-syn have been evaluated as potential biomarkers in PD diagnosis, disease monitoring, and clinical trial design.

### 2.1 Early diagnosis of PD and differentiation from other disorders

#### 2.1.1 Total alpha-synuclein

**Figure 1:**
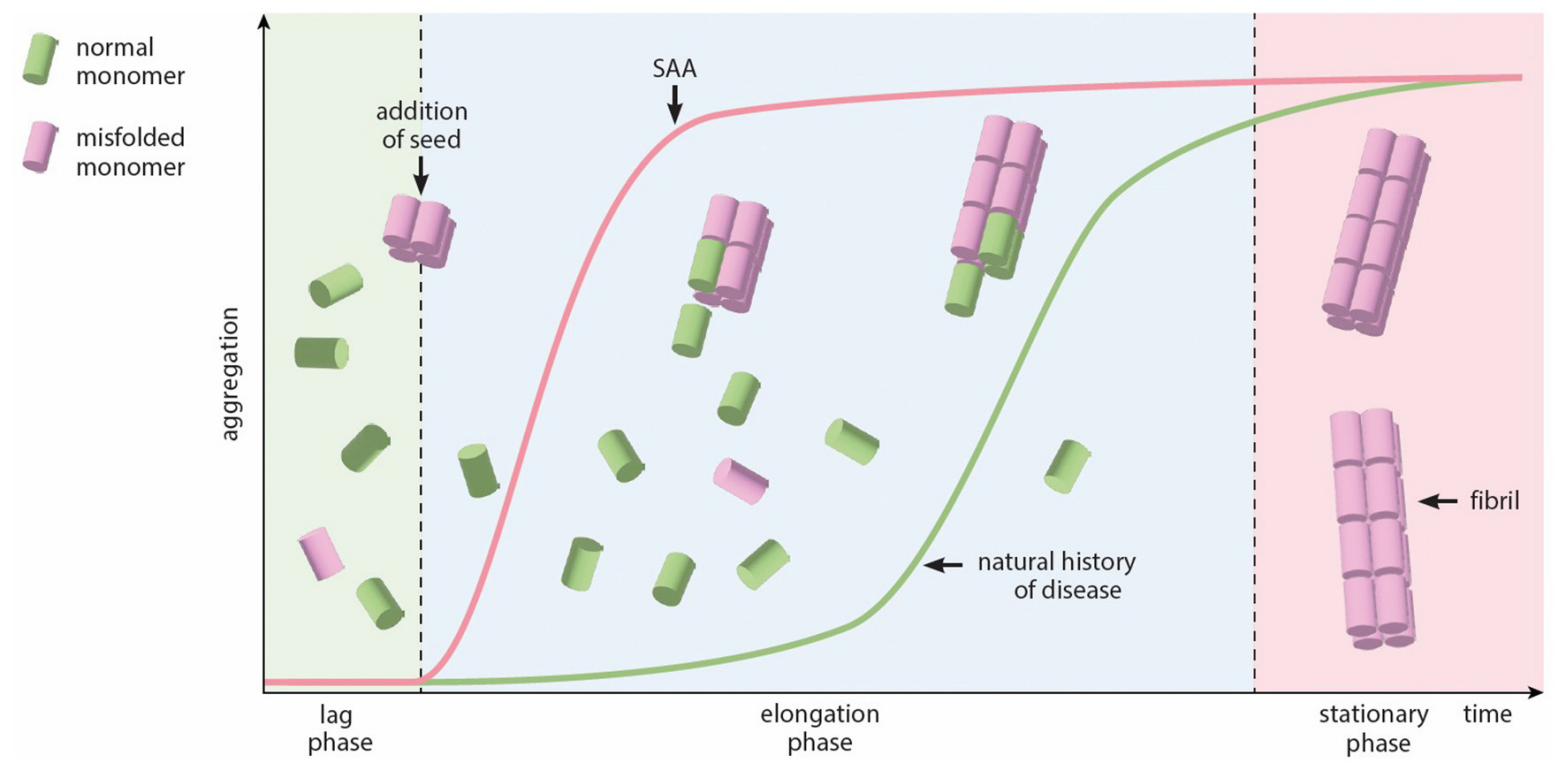
Schematic representation of the seeding/nucleation paradigm of α-synuclein misfolding and aggregation in the seed amplification assay (SAA). Early misfolding events leading to nucleus formation occur during the lag (nucleation) phase without detectable aggregation. Once a critical number of nuclei is formed, rapid aggregate growth ensues, resulting in the formation of protofibrils and fibrils. This is followed by the stationary phase, during which aggregation slows due to substrate depletion, reaching a dynamic equilibrium. In SAA, the addition of preformed seeds bypasses nucleus formation and significantly shortens the lag and elongation phases (blue curve vs. red curve), enabling earlier detection. In human biological samples such as blood, endogenous α-syn seeds undergo elongation and fragmentation, driving accelerated aggregation and fragmentation even in the absence of spontaneous aggregation. During quiescent incubation at 37 °C, elongation is followed by fragmentation, which increases the number of active seeding species. This cyclic process converts recombinant α-syn into aggregated forms, which are repeatedly fragmented, thereby amplifying the misfolded protein signal. By the end of the assay, most of the recombinant protein is incorporated into *in vitro*-generated aggregates, representing the bulk of total aggregates and enabling detection by conventional methods such as thioflavin T fluorescence. This schematic represents a simplified and integrated version of Figure 2A in Pinotsi et al., 2013, and Figure 1A in Concha-Marambio et al., 2023. This image has been reproduced from the book Parkinson's, Chapter 14 (In Search of Biomarkers for Parkinson's Disease in Biological Fluids, written by Goran Šimić), edited by Petra Bago Rožanković (ISBN: 978-953-191-054-5), with permission from the publisher (Naklada Slap, Jastrebarsko, Croatia).

Clinical heterogeneity among enrolled patients and methodological differences contribute to the limited diagnostic accuracy of measuring CSF total α-syn (t-α-syn), with low potential to differentiate PD from other neurodegenerative disorders (Førland et al., 2020; Schulz et al., 2021; Foulds et al., 2012; Twohig et al., 2018; Magdalinou et al., 2015; Mollenhauer et al., 2017; Goldman et al., 2018; Abdi et al., 2021; Zhou et al., 2015). Evaluation of *t*-α-syn in plasma, serum, or saliva as a biomarker for PD diagnosis has yielded conflicting results (De Bartolo et al., 2023; Chahine et al., 2020; Youssef et al., 2021). These findings reflect a high inter-study variability due to *inadequate control for pre-analytical factors* (sampling protocols, CSF blood contamination), *lack of standardized quantification methods* (assays, antibodies), and *unaddressed clinical heterogeneity* (disease stage, control group variability), leading to insufficient sensitivity and specificity (Ganguly et al., 2021; Hatano et al., 2024). A meta-analysis of 32 studies found a significant increase in plasma/serum *t*-α-syn levels in PD patients compared with healthy controls, with a stronger effect observed in the early stages of the disease (Zubelzu et al., 2022). Quantitative evaluation of plasma/serum α-syn levels has been performed using monoclonal antibodies coupled with magnetic nanoparticles, showing that both plasma and serum α-syn levels were higher in PD patients than in healthy controls (Chang et al., 2020). Recently introduced α-syn seed amplification assays (SAA) have shown high sensitivity (>95%) and specificity (98%) in detecting pathological α-syn aggregates (Rossi et al., 2020; Groveman et al., 2018). This approach is based on the ability of α-syn seeds to induce the formation of pathogenic oligomeric and fibrillary α-syn using real-time quaking-induced conversion (RT-QuIC) and protein misfolding cyclic amplification (PMCA) methods, as shown in Figure 1. (Fairfoul et al., 2016; Poggiolini et al., 2022; Wang et al., 2022). Results from a cross-sectional study evaluating CSF α-syn SAAs in a large cohort of 1,123 subjects demonstrated their feasibility in detecting PD compared to healthy controls (sensitivity 88%, specificity 96.3%) (Siderowf et al., 2023). Additionally, a positive CSF α-syn SAA result was found in 88% of subjects with hyposmia or rapid eye movement (REM) behavior disorder (RBD), suggesting its potential use in detecting the prodromal stage of PD (Siderowf et al., 2023). Positive CSF α-syn SAA results are more common in subjects at risk for PD, with the highest sensitivity observed in patients with glucocerebrosidase (*GBA1*) variants (96%) and the lowest in those with leucine-rich repeat kinase 2 (*LRRK2* Gly1920Ser) variants (68%) (Siderowf et al., 2023). Several other studies have also reported lower rates of positive α-syn SAA results in PD patients with *LRRK2* variants compared with those with *GBA1* variants (Rossi et al., 2020; Garrido et al., 2019; Brockmann et al., 2021). Additionally, CSF α-syn SAA results were negative in patients with *PRKN* and *PINK1* variants. Together with findings that these forms are associated with little or no Lewy body pathological changes, it has been suggested that CSF α-syn SAA may be more useful in PD with pronounced α-syn pathology (Brockmann et al., 2021; Poulopoulos et al., 2012; Henderson et al., 2019). This marker could help differentiate PD from progressive supranuclear palsy (PSP) and corticobasal degeneration (CBD), but with lower accuracy in differentiating PD from multiple system atrophy (MSA) (Grossauer et al., 2023; Wang et al., 2012). Recently, α-syn SAA activity has been classified based on signal intensity and association with different pathologies, showing specificity of 87% for MSA, 97% for PD, and 100% for idiopathic RBD (Ma et al., 2024; Wang et al., 2022).

A positive serum α-syn SAA result was found in all PD patients, even 1–10 years before clinical diagnosis, compared to negative SAA results in healthy controls (Kluge et al., 2024). In another study, plasma α-syn levels were significantly higher in PD patients than in healthy controls, with higher levels observed in patients with PD dementia (PDD) compared with those with mild cognitive impairment in PD (PD-MCI) or PD with normal cognition (Lin et al., 2017). Using the combined RT-QuIC technique with immunoprecipitation (IP/RT-QuIC), Okuzumi and colleagues evaluated the presence of α-syn in serum, reporting positive results in 95% of patients with PD, 90% of patients with dementia with Lewy bodies (DLB), and 44% of patients with RBD (Okuzumi et al., 2023). A recent study evaluating α-syn SAA in tear fluid reported positive results in 55% of PD patients, consistent with findings obtained from CSF (Canaslan et al., 2026). Results from several studies show that salivary *t*-α-syn levels are significantly lower in patients with PD than in healthy controls, while others failed to confirm these findings (Al-Nimer et al., 2014; Sabaei et al., 2023; Shaheen et al., 2020; Vivacqua et al., 2016, 2019). Wang et al. performed a prospective study on serum and saliva samples of PD patients and healthy controls using α-syn -SAA analyses, demonstrating high sensitivity (95.83%), specificity (96.15%), and accuracy (0.98) of combined serum and saliva α-syn-SAA with a potential for accurate PD diagnosis in clinical practice (Wang et al., 2024). Measuring *t*-α-syn alone cannot discriminate between physiological and pathological forms. In a healthy state, α-syn is present in high amounts in monomeric or tetrameric forms, which, during the neurodegenerative process, transform into pathogenic conformations, including oligomers and fibrils, post-translational modifications such as phosphorylated forms, or α-syn localization within exosomes (Giampa et al., 2021; Oueslati, 2016).

#### 2.1.2 Pathogenic forms of alpha-synuclein

Modification of physiological α-syn proteins into misfolded oligomeric forms represents a dominant pathological change in PD (Oueslati et al., 2010). Most studies show increased levels of oligomeric forms of α-syn (o-α-syn) and an increased o-α-syn/*t*-α-syn ratio in the CSF of PD patients compared to non-PD subjects (Tokuda et al., 2010; Hansson et al., 2014; Park et al., 2011; Cariulo et al., 2019; Majbour et al., 2016). Levels of o-α-syn are also increased in other fluids, including plasma (El-Agnaf et al., 2006; Duran et al., 2010), saliva and tears (De Bartolo et al., 2023; Kang et al., 2016; Vivacqua et al., 2019; Devic et al., 2011; Kharel et al., 2022; Hamm-Alvarez et al., 2019; Maass et al., 2020) and red blood cells (Papagiannakis et al., 2018; Daniele et al., 2018) in patients with PD, demonstrating significant diagnostic accuracy of serum o-α-syn (sensitivity of 75% and specificity of 100%) (Williams et al., 2016). Higher levels of oligomeric α-syn (o-α-syn) and the *o*-α-syn/t- α-syn ratio in the saliva of PD patients compared to controls have been reported (Devic et al., 2011; Al-Nimer et al., 2014; Vivacqua et al., 2016; Shaheen et al., 2020). A meta-analysis revealed that *o*-α-syn in saliva has the potential to become a diagnostic biomarker in PD (Bougea et al., 2019). However, results from some studies have shown decreased levels of *o*-α-syn in PD samples or no significant differences compared to healthy controls (Park et al., 2011; Yanamandra et al., 2011; Wang et al., 2015). Recently, a novel saliva biomarker panel composed of o-α-syn, t-tau, microtubule-associated protein light chain 3 beta (MAP-LC3β), and tumor necrosis factor alpha (TNF-α) showed higher levels in *de novo* PD patients than in healthy controls (De Bartolo et al., 2023). The combined measurement of the ratios of o-α-syn/t-α-syn and amyloid-β (Aβ-42)/total-tau improves the differentiation of PD from tauopathies (Meloni et al., 2023). PMCA-based evaluation of o-α-syn could differentiate MSA from PD/DLB patients (Rossi et al., 2020; Singer et al., 2020). There have been attempts to evaluate o-α-syn in erythrocytes as a potential biomarker in PD. Results from a recent large study showed that levels of o-α-syn in erythrocytes were higher in PD patients than in patients with MSA and healthy controls (Li et al., 2019). However, most recent studies have not confirmed o-α-syn as a biomarker for differentiating PD from other neurodegenerative disorders (Eusebi et al., 2017; Majbour et al., 2020; Luo et al., 2024).

Post-translational phosphorylation modifies α-syn, contributing to its misfolding and aggregation (Oueslati et al., 2010). The primary modified form of α-syn is phosphorylation at serine 129 (pS129-α-syn), which represents the main target in biomarker studies of phosphorylated forms of α-syn (p-α-syn) (Anderson et al., 2006). Most studies have reported elevated levels of CSF pS129-α-syn in PD compared to controls, although some studies did not reach statistical significance (Majbour et al., 2016; Oosterveld et al., 2020). Similar levels of CSF pS129-α-syn were observed in atypical parkinsonian syndromes, limiting its use in differential diagnosis (van Steenoven et al., 2018; Foulds et al., 2012; Majbour et al., 2020). However, results from several studies have shown higher levels of CSF pS129-α-syn in PD compared to MSA patients, although this finding was not confirmed in other studies (Wang et al., 2012; Schulz et al., 2021; Foulds et al., 2012; Constatidines et al., 2021; Cao et al., 2020). Several reports have questioned the potential of this biomarker in differentiating PD from tauopathies, including PSP, CBS, and AD (Schulz et al., 2021; Foulds et al., 2012; Constatidines et al., 2021; van Steenoven et al., 2018; Swallow and Counsell, 2023). Results of a recent study reported lower levels of t-α-syn and higher levels of o-α-syn and p-tau in PD patients compared to healthy controls (Daniele et al., 2018). A promising diagnostic marker for PD may be the combination of different α-synforms, particularly an increased ratio of Ser-129-p-α-syn and/or o-α-syn to t-α-syn (Zheng et al., 2021; Parnetti et al., 2014; van Steenoven et al., 2018; Majbour et al., 2020; Stewart et al., 2015).

### 2.2 Disease progression and α-syn

Available data on CSF t-α-syn levels throughout the PD course show contradictory results, since α-syn is aggregated in Lewy bodies but also released from degenerating synapses (Majbour et al., 2016; Hall et al., 2026; Mollenhauer et al., 2019; Paoletti et al., 2020). Most studies suggest that t-α-syn measured in CSF, blood, and EVs has a limited ability to predict and track PD progression (Mollenhauer et al., 2011; Eusebi et al., 2017; Wang et al., 2018; Stuendl et al., 2016; Shi et al., 2014; Niu et al., 2020; Jiang et al., 2020; Parnetti et al., 2019; Constatidines et al., 2021). CSF t-α-syn does not change over time in relation to motor or cognitive symptoms (Mollenhauer et al., 2017; Hall et al., 2026). However, a few studies have suggested that higher CSF t-α-syn correlates with greater PD progression as a manifestation of more pronounced neurodegeneration (Hall et al., 2015). Kang et al. observed a significant decrease in the o-α-syn/t-α-syn ratio in Hoehn and Yahr stage I (H&Y stage I) PD patients and a significant increase in the ratio in HY stage II-IV PD patients, suggesting a possible role as a biomarker of PD progression (Kang et al., 2016).

CSF o-α-syn levels increased at a 4-year follow-up in the PD group and correlated with motor progression (Majbour et al., 2021), particularly with postural instability and gait difficulties (Majbour et al., 2016). Despite some conflicting results, most studies suggest that CSF o-α-syn predicts PD progression (Russo et al., 2021; Majbour et al., 2016, 2021).

Results from a prospective study evaluating 122 PD patients and 68 controls, followed for 3 years, showed that plasma levels of t-α-syn and pS129-α-syn were significantly higher in PD patients than in controls, and pS129-α-syn levels were higher in patients with advanced motor symptoms. PD patients with a specific baseline pS129-α-syn level (> 8.5 fg/ml) experienced greater motor progression than those with lower pS129- α-syn levels (<8.5 fg/ml, *p* = 0.03) (Lin et al., 2019a). Results from some other studies confirm the correlation of pS129-α-syn levels with the progression of motor symptoms (Stewart et al., 2015; Majbour et al., 2016). However, some studies have reported lower pS129-α-syn levels in later disease stages, possibly because of extensive neuronal damage (Majbour et al., 2021; Foulds et al., 2012).

## 3 Alzheimer's disease-related biomarkers

### 3.1 Differentiating Parkinson's disease from other disorders

Studies have shown that amyloid plaques and neurofibrillary tangles can coexist with α-syn pathology in PD (Jellinger K, 2008; Compta et al., 2011; Irwin et al., 2013; Jellinger et al., 2002; Williams et al., 2016). A combination of reduced Aβ-42 and increased total tau (t-tau) and phosphorylated tau (*p*-tau), known as an AD biomarker profile, can occur in synucleinopathies, including PD, in patients with cognitive decline (Kurata et al., 2007; Clinton et al., 2010). Current data show a low diagnostic value of CSF Aβ-42 in PD patients (Kang et al., 2013; Stav et al., 2015; Delgado-Alvarado et al., 2016; Kang et al., 2016). However, most studies demonstrate that lower CSF levels of Aβ-42 are related to worse cognitive function in PD patients (Irwin et al., 2020, 2017; Parnetti et al., 2014; Alves et al., 2014; Kang et al., 2013; Montine et al., 2010; Compta et al., 2013). Patients with dementia with Lewy bodies (DLB), AD, and PDD presented with lower levels of CSF Aβ-42 compared to PD patients without dementia (Vranová et al., 2014; Kaerst et al., 2013). In another study, the CSF AD-specific biomarker profile was found in a higher percentage of patients with DLB (25%) compared to only 3% of patients with PD (van Steenoven et al., 2016).

Studies evaluating t-tau and p-tau in CSF show contradictory results (Alves et al., 2014; Hu et al., 2017; Chiu et al., 2021). A recent study demonstrated higher serum p-tau levels in PD compared to healthy controls and in PD with cognitive impairment compared to PD without cognitive impairment (Blommer et al., 2023).

### 3.2 Predicting disease progression and phenotype

Decreased levels of Aβ-42 in combination with increased levels of t-tau and p-tau in CSF have been correlated with greater progression of cognitive decline in PD and AD (Berlyand et al., 2016; Leaver and Poston, 2015; Siderowf et al., 2010; Henderson et al., 2019). Available data show that PD patients with lower CSF Aβ-42 levels at disease onset have a more rapid progression to dementia (Parnetti et al., 2014; Siderowf et al., 2010; Alves et al., 2014), suggesting that this biomarker may serve as a prognostic factor for the development of cognitive impairment (Blennow et al., 2016). An AD-related biomarker profile in PD patients has been correlated with faster progression to dementia, greater memory deficits, and the akinetic-rigid disease form (Irwin et al., 2017; Compta et al., 2011; Halliday et al., 2008; Howlett et al., 2015; Sabbagh et al., 2009; Coughlin et al., 2019). However, other studies failed to confirm more pronounced AD biomarkers in the diffuse-malignant subtype compared to the mild motor dominant subtype of PD (Fereshtehnejad et al., 2017, 2015). In a prospective study evaluating PD patients for 3 years, lower initial CSF Aβ-42 levels predicted cognitive and motor decline, autonomic dysfunction, and dopa-resistant gait impairments (Rochester et al., 2017). A few studies have demonstrated that p-tau181 levels could predict disease progression and cognitive decline (Batzu et al., 2022; Chiu et al., 2021; Pagonabarraga et al., 2022).

## 4 Neuroinflammation

In recent years, an increasing number of studies have evaluated various inflammatory mediators as potential biomarkers in PD, given the evidence that immune cells in the central nervous system (CNS) and peripheral blood are involved in PD pathogenesis (Ransohoff, 2016; Caldi Gomes et al., 2022; Nayak et al., 2015; Dixit et al., 2019; Magdalinou et al., 2017; Kouli and Williams-Gray, 2020). Microglia, which represent the primary innate immune cells of the CNS, can be activated by misfolded proteins and protein aggregates, thereby inducing neuroinflammation (de Araujo et al., 2021). Different proteins, including α-sin, act as epitopes that activate the immune response in peripheral blood mononuclear cells (PBMCs) in patients with PD (Sulzer et al., 2017; White et al., 2018). Inflammatory processes in PD appear to be most prominent in the early stages of the disease and may persist throughout disease progression (Lindestam Arlehamn et al., 2020).

Proteins released from astrocytes measured in blood have been evaluated in PD and other neurodegenerative disorders. Glial fibrillary acidic protein (GFAP) levels were elevated in PD patients compared to healthy controls, but this marker was unable to differentiate PD from atypical parkinsonian syndromes (Su et al., 2012; Oeckl et al., 2019). Chitinase-3-like protein 1 (YKL-40) and monocyte chemoattractant protein-1 (MCP-1) were significantly higher in tauopathies than in PD (Olsson et al., 2013; Wennstrom et al., 2015).

Results from studies evaluating inflammatory markers in blood have shown that IL-1β and CRP, and to a lesser extent IL-2, IL-6, IL-8, IFN-γ, and TNF-α, may have diagnostic value in PD (Hu et al., 2015; Miliukhina et al., 2020; Rocha et al., 2018; Lian et al., 2019; Chatterjee et al., 2020; Fan et al., 2020; Schroder et al., 2018; Delgado-Alvarado et al., 2017; Miliukhina et al., 2020; Gupta et al., 2016; Calvani et al., 2020; Usenko et al., 2020; Eidson et al., 2017; Martin-Ruiz et al., 2020; Qiu et al., 2019). Elevated levels of CRP, IL-6, IL-10, TNF-αand chemokine (C-C motif) ligand 5 (CCL5) have also been reported in PD patients (Jin et al., 2020; Qiu et al., 2019; Vesely et al., 2018; Karpenko et al., 2018; Kim et al., 2018; Kouchaki et al., 2018). A few studies have evaluated CSF inflammatory markers, showing higher levels of IL-1β in PD patients compared to healthy controls (Hu et al., 2015; Chen et al., 2018; Iwaoka et al., 2020). Some studies have reported elevated CSF levels of IL-6 and IL-8 in PD patients, although these results were not consistently replicated in other studies (Chen et al., 2018; Iwaoka et al., 2020; Lian et al., 2019; Schroder et al., 2018; Hall et al., 2018; Hu et al., 2019; Santaella et al., 2020; Delgado-Alvarado et al., 2017). There are reports that higher blood levels of IL-10 are associated with greater motor and cognitive impairment (Usenko et al., 2020; Martin-Ruiz et al., 2020). Other studies have shown that higher blood levels of IL-1β, IL-15, IFN-γ, S100B, TNF-α, and VCAM1, together with lower levels of anti-inflammatory cytokines IL-4 and IL-12p40, are associated with worse motor function in PD patients (Eidson et al., 2017; Fan et al., 2020; Lerche et al., 2022; Perner et al., 2019; Yilmaz et al., 2018). A few longitudinal studies have reported higher blood levels of CRP, C3, and C4 in PD patients with cognitive decline (Mollenhauer et al., 2019; Vesely et al., 2019). Several cross-sectional studies have demonstrated that higher CSF levels of CRP, IL-6, MCP-1, FABP, IL-8, and YKL-40 are associated with worse cognitive function, and that higher levels of CRP and MCP-1 are associated with depression and fatigue in PD patients (Hall et al., 2018; Sanjari Moghaddam et al., 2018; Lindqvist et al., 2013).

However, variability in patient cohorts, differences in diagnostic methods, and gender-related differences in immune responses contribute to the inconsistent findings across studies evaluating different immune parameters in PD.

## 5 Extracellular vesicles

Extracellular vesicles (EVs), small particles released from many cell types, have been investigated as potential biomarkers for diagnosing different neurodegenerative disorders, including PD (Quek and Hill, 2017; Latifkar et al., 2019; Boing et al., 2014). They are composed of exosomes (50-150 nm), microvesicles (100–1000 nm), and apoptotic bodies (up to 5 μm) (Agliardi et al., 2021). Different exosomal proteins, such as α-syn, inflammation-related markers, AD-related markers, and microRNA (miRNA), have been investigated as potential biomarkers for PD (Keighron et al., 2023; Hook et al., 2023; Margis et al., 2011).

### 5.1 Pathological proteins in exosomes

Neuron-derived extracellular vesicles (NDEVs) containing α-syn can cross the blood-brain barrier and can therefore be detected in peripheral fluids (Shi et al., 2014; Ishiguro et al., 2024). Results from a recently published meta-analysis showed a significant increase in α-syn levels detected in both total exosomes and neuron-derived exosomes from patients with PD compared with healthy controls (Kim et al., 2024). Another meta-analysis by Xylaki and colleagues confirmed that the detection of α-syn could serve as a potential biomarker for diagnosing PD (Xylaki et al., 2023). Reported data suggest that measurement of t-α-syn in EVs from different biological fluids, including CSF, plasma/serum, or saliva, can distinguish PD from healthy controls (Stuendl et al., 2016, 2021; Zhao et al., 2019; Jiang et al., 2020; Niu et al., 2020; Dutta et al., 2021; Fu et al., 2020; Cao et al., 2019; Si et al., 2019; Zhao and Yang, 2021; Ohmichi et al., 2019; Xylaki et al., 2023; Church et al., 2024). There were no significant differences in pS129-α-syn in EVs compared with its levels in whole plasma in patients with PD. However, recent studies have reported that levels of pS129-α-syn in the cytosolic fraction of erythrocytes are significantly higher in PD patients than in controls (Tian et al., 2019; Foulds et al., 2012), even in the earliest stages of the disease (Li P. Y. et al., 2021). Another study did not confirm these findings (Chatterjee et al., 2020).

Studies performed on other biological fluids have shown elevated EV levels of p-Ser-1292 LRRK2 in urine from patients with PD (Wang et al., 2017; Fraser et al., 2016). Rani et al. (2019) confirmed higher neuronal EV α-syn levels in saliva, suggesting its potential as a PD biomarker. Recent results from a longitudinal study demonstrate an increase in both t-α- Syn and o-α- Syn in PD- derived salivary EVs compared to healthy controls, with higher levels observed in patients with cognitive decline and motor fluctuations. There were no correlations with H&Y stage, and no change in α-syn levels after 1 year of follow-up (Gurgone et al., 2026). Cao et al. evaluated different forms of α-syn in saliva EVs in PD patients, showing increased levels of o-α-syn and o-α-syn/t-α-syn ratios with no significant differences in p-α-syn and p-α-syn/t-α-syn ratios compared to healthy controls (Cao et al., 2019). The results from another study, using Nanoparticle Tracking Analysis (NTA), revealed a higher concentration of the fluorescence-dye-labeled salivary EVs in PD compared to controls (*p* = 0.0001) with higher levels of t-α-syn, p-α-syn/t-α-syn ratios in PD patients, and a significant increase in t-α-syn in prodromal PD patients (Rastogi et al., 2023).

Although EVs offer a window into CNS-derived cargo, several methodological challenges limit their current clinical utility. Variability in isolation methods (ultracentrifugation vs. precipitation vs. immunocapture), differences in EV subtypes enriched (L1CAM-based vs. non-enriched neuronal EVs), and pre-analytical factors (processing time, temperature, hemolysis) can drastically alter measured α-syn levels in EVs. Saliva EVs deserve particular scrutiny: several studies report altered α-syn species in salivary EVs, but results are not uniformly replicated across cohorts. The study of Gurgone et al. provides important insights but also highlights the need for standardization and multi-cohort validation (Gurgone et al., 2026). Interpretation should be cautious: elevated EV-associated α-syn in saliva/plasma may reflect peripheral release, BBB/transporter dynamics, or peripheral mimicry of CNS pathology. Therefore, claims of disease specificity require replication in independent samples and cross-validation with CSF and neuroimaging data. A critical objective going forward is to establish consensus protocols for EV isolation from plasma, CSF, and saliva. EV isolation protocols from plasma are more extensively studied, with several established methods such as ultracentrifugation, size exclusion chromatography, and precipitation-based approaches available. However, a universally accepted protocol for purifying plasma-derived EVs is still lacking, as none of the aforementioned methods is considered the “gold standard” (Takov et al., 2018; Veerman et al., 2021; Welsh et al., 2024; Zanganeh et al., 2026). In contrast, while salivary EV isolation and analysis methods are still under development, there is progress in methodological comparisons being published, and the latest version of MISEV guidelines (MISEV2023) includes a dedicated section on saliva (Welsh et al., 2024).

There is also a need to integrate RT-QuIC/PMCA findings with EV cargo data to determine whether EV-associated seeds correlate with seeding activity in the same samples, potentially strengthening diagnostic specificity.

Measurement of α-syn levels in NDEVs may contribute to better differentiation of PD from other atypical parkinsonian syndromes, especially when combined with levels of other proteins such as clusterin (Jiang et al., 2020; Dutta et al., 2021; Jiang et al., 2021). Evidence from recent studies suggests that α-syn derived from NDEVs in blood may even predict the development of PD in patients at risk, including those with symptoms of RBD, hyposmia, or non-manifesting carriers of *GBA* mutations (Yan et al., 2024).

Regarding other proteins, plasma EV levels of tau and Aβ-42 have been found to be significantly increased in PD patients compared with healthy controls, particularly in relation to cognitive impairment (Chan et al., 2023; Parnetti et al., 2019). Elevated levels of Aβ-42 and tau, together with increased α-syn and lower levels of serine-phosphorylated insulin receptor substrate (IRS-p312) in serum EVs, predict worse motor and cognitive progression in PD patients (Chung et al., 2021; Chan et al., 2023; Blommer et al., 2023). Plasma exosomal tau may differentiate PD from healthy controls but not from AD (Shi et al., 2016). Among chemokines in extracellular fluids that regulate inflammation and immune responses, C-X-C motif chemokine ligand 12 (CXCL12) in plasma exosomes has been proposed as a potential biomarker for diagnosing PD (Bagheri et al., 2018; Li et al., 2022). Results from another study revealed that CXCL12 levels correlated with autonomic dysfunction, while α-syn levels in NDEVs correlated with clinical stage as well as motor and non-motor symptoms in PD (Jiang et al., 2020).

However, technical challenges, including inadequate controls for the isolation of neuronal extracellular vesicles and variability in α-syn forms, contribute to the lack of protocol standardization and limit their applicability in clinical practice (Bernhardt et al., 2024; Kluge et al., 2024; Norman et al., 2021).

Based on these studies, it can be concluded that the field of saliva EV biomarkers is promising and rapidly evolving, with methodological comparisons being published and the latest version of the MISEV guidelines (MISEV2023), including a dedicated section on saliva (Welsh et al., 2024). Future research is warranted to characterize the cargo of salivary EVs, to determine their potential for early diagnosis and disease monitoring, and to explicitly report normalization strategies and quality controls to improve comparability. Moreover, where α-syn within EVs is detected, parallel RT-QuIC testing of the same biofluid can help determine whether EV cargo reflects seed-competent species. This integration has the potential to improve diagnostic specificity for PD vs. atypical parkinsonian disorders. Finally, it is also of high clinical relevance to emphasize that the strongest translational signal currently comes from multi-modal panels (EV cargo + inflammatory markers + AD biomarkers) rather than single analytes. To establish clinical utility, the field should therefore prioritize multi-marker, cross-fluid validation in longitudinal cohorts.

### 5.2 MicroRNA in exosomes

MicroRNAs are small non-coding RNAs with 20–22 nucleotides that play a role in the pathogenesis of different neurodegenerative disorders, including PD (Maiese, 2022; Guévremont et al., 2023; van Wamelen et al., 2020). Available data suggest that certain microRNAs in plasma exosomes, including miR-24, miR-23b-3p, miR-29c, miR-195-3p, and miR-331-5p, have potential as biomarkers for PD (Harischandra et al., 2018). Among microRNAs, miR-30c and miR-148b were reported to be specific to PD, while miR-24, miR-34b, and miR-148b were more prevalent in MSA compared to PD (Villar-Menéndez et al., 2014; Vallelunga et al., 2014). In another study, downregulation of miR-19b and upregulation of miR-195 and miR-24 demonstrated accuracy ranging from 70 to 90% in differentiating PD from healthy controls. Several studies evaluating different microRNAs in saliva have shown variable results. Results from the recent study show decreased levels of miR-153 and miR-223 in the saliva of PD patients, with no correlation with H&Y stage (Cressatti et al., 2020). In another study, miR-29a-3p and miR-29c-3p were decreased, while miR6765-5p was increased in PD patients, with further results showing the potential of miR-29 to differentiate PD from healthy controls, and miR-29a-3p PD from essential tremor (ET) and multiple system atrophy (MSA) (Jiang et al., 2020). Evaluation of miRNA in plasma EVs revealed upregulation of miR-151a-5a, let-7e-5p, miR-9-5p, miR-127-3p, and miR-146a-5p, and downregulation of miR-93-5p (Nie et al., 2020; Sproviero et al., 2021; Li et al., 2024) in PD patients. Results from another study evaluating miRNA in serum EVs showed decreased levels of let-7i-5p, miR-18a-5p, and miR-20a-5p, and increased levels of miR-143-3p, miR-151a-3p, and miR-151a-5p in PD (Miyamoto et al., 2022).

From the results of the aforementioned studies, it can be concluded that microRNAs in NDEVs offer complementary information to protein cargo and may reflect CNS-derived regulatory networks. However, normalization, platform choice (qRT-PCR vs. sequencing), and hemolysis remain major sources of variability that hinder cross-cohort replication. Thus, while key miRNA candidates show promise in certain cohorts, external validation is needed. Once external validation experiments are completed (including the usage of synthetic spike-ins, exogenous controls, and assessment of the impact of hemolysis on plasma/serum miRNA profiles), multi-omic EV signatures combining miRNA and protein cargo, anchored by robust normalization and cross-platform harmonization, would strengthen translational potential. Table 1 provides an integrated view of the diagnostic and prognostic strength across major biomarker classes in PD biofluids; Table 2 compares biomarkers across fluids, and Table 3 lists saliva EV studies.

**Table 1:** A cross-class synthesis of major biomarkers in PD biofluid analysis.

Biomarker class	Representative biomarkers	Fluids (compartments)	Evidence type	Study designs	Strength of evidence	Key references	Notes
α-syn species	total α-syn (t-α-syn); oligomeric α-syn (o-α-syn); phosphorylated α-syn (pS129-α-syn)	CSF; plasma/serum; saliva	Observational studies; secondary analyses; some SAA data	Cross-sectional; longitudinal	Low - High	Majbour et al., 2016; Rossi et al., 2020; Siderowf et al., 2023; van Steenoven et al., 2018	Results should be interpreted cautiously due to assay/cohort heterogeneity
SAA/RT-QuIC (seed amplification)	α-syn seed amplification assays (RT-QuIC; PMCA)	CSF; serum/plasma; saliva	Diagnostic assay data; prodromal signals	Cross-sectional; some longitudinal	High	Rossi et al., 2020; Siderowf et al., 2023; Okuzumi et al., 2023; Poggiolini et al., 2022	Requires standardization and broader validation
Neurofilament light chain (NfL)	NfL in CSF and blood	CSF; plasma/serum	Diagnostic and prognostic biomarker data	Cross-sectional; longitudinal	Moderate—High	Bridel et al., 2019; Lin et al., 2019a; Ma et al., 2021	Confounded by age and comorbidities; best in panels
Glial fibrillary acidic protein (GFAP)	GFAP in serum/plasma	Blood	Prognostic biomarker data	Cross-sectional; longitudinal	Moderate	Su et al., 2012; Lin et al., 2023; Tang et al., 2023	More informative when combined with other markers
YKL-40	YKL-40 in CSF	CSF	Differential biomarker data across neurodegenerative disorders	Cross-sectional	Moderate	Wennstrom et al., 2015; Hall et al., 2018	Not PD-specific; more informative for differential diagnosis
AD biomarkers	(Aβ42, total-tau [t-tau], phospho-tau [p-tau])	CSF; blood	Differential diagnosis; PD with cognitive impairment	Cross-sectional; longitudinal	Moderate	Irwin et al., 2017; Rochester et al., 2017; Halliday et al., 2008	Useful for risk stratification in relation to cognitive changes in PD
Exosomal miRNAs	miR-24; miR-29c; miR-195; mir-331-5p; others	Plasma exosomes; saliva exosomes	Diagnostic/prognostic signals	Cross-sectional; longitudinal	Low-Moderate	Margis et al., 2011; Villar-Menéndez et al., 2014; Guévremont et al., 2023	High methodological variability; replication needed
EV cargo in NDEVs (α-syn, tau, Aβ; miRNA)	α-syn in NDEVs; tau/Aβ cargo; exosomal miRNA	Plasma exosomes; CSF exosomes	Diagnostic/prognostic	Cross-sectional; longitudinal	Moderate	Shi et al., 2014; Jiang et al., 2020; Chan et al., 2023	Promising but requires standardization

**Table 2:** PD biomarkers: cross-fluid performance.

Biomarker	Fluid(s) tested	Study designs (cross-sectional/ longitudinal)	Prognostic/ progression data	Population/ comparison groups	Key references	Notes
α-syn SAA/RT-QuIC	CSF; plasma/serum; saliva	Cross-sectional; longitudinal	Progression associations	PD vs controls; PD vs DLB/MSA; prodromal groups	Rossi et al., 2020; Siderowf et al., 2023; Okuzumi et al., 2023; Poggiolini et al., 2022	Assay subtypes may differ as well as sample handling
Total α-syn (t-α-syn)	CSF; plasma	Cross-sectional; longitudinal	Progression associations	PD vs. controls; PD subtypes	Førland et al., 2020; Foulds et al., 2012; Mollenhauer et al., 2011	Highly assay- and cohort-dependent
Neurofilament light chain (NfL)	CSF; plasma/serum	Cross-sectional; longitudinal	Cognitive/motor progression	PD vs MSA/PSP	Bridel et al., 2019; Lin et al., 2019a; Ma et al., 2021	Age-adjusted analyses recommended
Glial fibrillary acidic protein (GFAP)	Serum/plasma	Cross-sectional; longitudinal	Cognitive progression signals	PD vs controls; conversion risk	Lin et al., 2023; Tang et al., 2023	Best in combination panels
YKL-40	CSF	Cross-sectional	Cognitive associations	PD vs. AD/tauopathies	Wennstrom et al., 2015; Hall et al., 2018	More informative for differential diagnosis
AD biomarkers (Aβ42, t-tau, p-tau)	CSF; serum	Cross-sectional; longitudinal	Cognitive impairment	PD with cognitive impairment (PDD); dementia risk	Irwin et al., 2017; Rochester et al., 2017	Useful for risk stratification in cognitive changes in PD
Exosomal miRNAs	Plasma exosomes; saliva exosomes	Cross-sectional; longitudinal	Cognitive/clinical correlations	PD vs. controls; MSA	Margis et al., 2011; Villar-Menéndez et al., 2014; Guévremont et al., 2023	High methodological variability; replication needed
EV cargo in NDEVs (α-syn, tau, Aβ; miRNA)	Plasma exosomes; CSF exosomes	Cross-sectional; longitudinal	Cognition, progression signals	PD vs controls; prodromal risk	Shi et al., 2014; Jiang et al., 2020; Chan et al., 2023	Promising but requires harmonized protocols

**Table 3:** Saliva EV studies.

Study (Author, Year)	Saliva EV cargo studies	Isolation method	Population/ comparison groups	Key findings	Limitations
Cao et al. (2019)	α-syn (t-/o- forms) in salivary EVs	Salivary EV isolation with differential centrifugation and affinity capture	PD vs controls	Elevated salivary EV α-syn	Small cohorts; heterogeneity in methods
Rani et al. (2019)	Neuronal exosomal α-synuclein in saliva	Neuronal marker-based enrichment in saliva EVs	PD vs. controls	Higher neuronal EV α-syn	Pilot study; replication needed
De Bartolo et al. (2023)	Salivary biomarker panel (contextual, not strictly EV)	Saliva collection; multi-omics readouts	Panel differentiates *de novo* PD from controls in initial cohorts	Integration with EV data not direct	Not strictly EV-focused
Gurgone et al. (2026)	α-syn (t-/o- forms) in salivary EVs in longitudinal study	Salivary EV isolation with differential centrifugation	PD vs controls	Elevated salivary EV α-syn; higher o-α-syn levels in cognitive decline and motor fluctuations; no change in 1-year follow-up	Small cohorts; short follow-up
Rastogi et al. (2023)	α-syn (t-/p- forms), CD9, CD63, flotillin-1, L1CAM in salivary EVs	Salivary EV isolation with differential centrifugation and ultrafiltration	PD vs controls	Elevated salivary EV t-α-syn; p-α-syn, p/t-α-syn ratio, CD9, CD63, flotillin-1, L1CAM; elevated t-α-syn in prodromal PD stage	Small cohorts

## 6 Non-specific biomarkers of neurodegeneration

### 6.1 Neurofilament light chain

Neurofilament light chain (NfL) is a protein released into CSF and blood following neuro-axonal damage and neurodegeneration (Khalil et al., 2024). Most studies have failed to provide evidence supporting NfL as a biomarker for differentiating PD from healthy controls, which could be attributed to confounding factors, including age, body mass index (BMI), and cardiovascular risks (85-89). Some studies have reported significantly higher serum NfL levels in PD patients than in patients with essential tremor (ET) and healthy controls (Liu et al., 2023; Hansson et al., 2017).

However, NfL shows potential as a biomarker for differentiating PD from atypical parkinsonian disorders. Higher CSF NfL levels have been reported in different atypical parkinsonian disorders, with excellent accuracy in distinguishing PD from PSP (sensitivity 93%, specificity 95%), MSA (sensitivity 89%, specificity 93%), and CBS (sensitivity 100%, specificity 93%) (Hansson et al., 2017; Herbert et al., 2015; Bridel et al., 2019).

Results from different studies have shown conflicting data regarding the association between NfL levels and motor symptoms in PD (Niemman et al., 2021; Oosterveld et al., 2020; Pötter-Nerger et al., 2022). Lin et al. (2019b) evaluated plasma NfL levels and and cognitive symptoms in 116 PD patients, 22 patients with MSA, and 40 healthy controls at baseline and after 3 years. Plasma NfL levels were significantly higher in MSA patients than in PD patients and controls, and higher in PD patients with more pronounced motor symptoms and dementia. Additionally, higher baseline NfL levels correlated with greater motor and cognitive progression. Other studies have also reported greater cognitive progression in PD patients with higher NfL levels measured in CSF or blood (Oosterveld et al., 2020; Ma et al., 2021; Lin et al., 2019a). These findings suggest that NfL may serve as a biomarker for distinguishing PD from atypical parkinsonian disorders and for predicting cognitive decline.

### 6.2 Glial fibrillary acidic protein

In CNS damage, astrocytes release the intermediate filament protein glial fibrillary acidic protein (GFAP), which can be measured in CSF and blood (Ishiki et al., 2016), (Liddelow and Barres, 2017). GFAP is not disease-specific, as its levels are increased in different disorders, including traumatic brain injury, cerebrovascular disorders, and neurodegenerative diseases (Lotankar et al., 2017). Serum GFAP levels have been reported to be higher in PD patients compared with healthy controls, with no significant difference observed between PD patients and those with acute stroke (Su et al., 2012). Results from recent studies have shown that baseline plasma GFAP levels may predict rapid progression to cognitive decline and postural instability (Lin et al., 2023; Liu et al., 2023; Lin et al., 2023; Tang et al., 2023). Results from other studies also suggest that GFAP may serve as a biomarker of progression to dementia in PD, showing higher levels in PDD compared with PD MCI and healthy controls (Bartl et al., 2023; Tang et al., 2023; Lin et al., 2023; Che et al., 2024). Other studies suggest that a combination of GFAP and neurofilament light chain (NfL) can differentiate PDD from PD without dementia (Mao et al., 2023) and correlates with the progression of motor symptoms (Youssef et al., 2023). Based on the available data, GFAP has potential as a biomarker of disease progression, but not as a diagnostic biomarker for differentiating PD from other conditions.

In summary, higher NfL levels track progressive neurodegeneration and cognitive decline across PD and related disorders, although specificity for PD remains a challenge in the absence of a PD-specific biomaker panel, GFAP and other glial markers may predict conversion to dementia and correlate with motor progression in longitudinal cohorts, while CSF Aβ42, total tau (t-tau), and phosphorylated tau (p-tau) are associated with faster cognitive decline and may identify patients at higher risk for PD dementia (PDD). On translational relevance, the biomarkers should always be evaluated for which ones demonstrate the most consistent associations with Hoehn–Yahr stages, UPDRS scores, and cognitive scales (MoCA, MMSE), and whether they can predict response to dopaminergic therapy or progression to dementia, highlighting the critical need for longitudinal, treatment-stratified cohorts to establish clinical utility and inform trial design.

## 7 Conclusion

This review summarizes current data on various biofluid biomarkers for PD, including α-syn variants, different proteins or microRNAs released from EVs, and markers associated with neuroinflammation and AD. While significant progress has been made in identifying potential biomarkers in various biofluids, including plasma, CSF, and saliva through advances in the detection of pathological α-syn using PMCA and RT-QuIC assays, which have provided new opportunities for the diagnosis of PD and its differentiation from other neurodegenerative disorders, the translation of these findings into clinical practice is hindered by several major challenges. These challenges include the lack of standardized methodologies for sample collection, processing, and analysis, as well as the need for longitudinal studies to assess the predictive and prognostic value of candidate biomarkers. Additionally, variability among studies, including differences in patient populations with respect to different stages and PD subtypes, the limited inclusion of patients with atypical parkinsonian syndromes, and technical issues such as sample contamination, have further limited the clinical applicability of these biomarkers.

Recent studies highlight the potential of NDEVs containing t-α-syn, pS129-α-syn, Aβ42, chemokines, and microRNAs as biomarkers for PD diagnosis, disease progression, and prediction of future disease development. The CSF AD biomarker profile has been associated with worse cognitive function in PD patients and may serve as a prognostic factor for the development of dementia. In contrast, markers of neuroinflammation have generally failed to demonstrate applicability for detecting or monitoring PD.

Salivary EVs, in particular, represent a promising, but still largely unexplored, source of biomarkers for PD. Further research is warranted to characterize the cargo of salivary EVs and to determine their potential for early diagnosis and disease monitoring. Integrating EV-based biomarkers with other diagnostic tools, such as RT-QuIC, may improve the accuracy and reliability of PD diagnosis.

Future research should prioritize large-scale, multi-center studies that incorporate standardized protocols and longitudinal follow-up with special emphasis on a multi-marker panel approach. These studies should also focus on correlating biomarker levels with clinical features of the disease, such as disease stage, motor and non-motor symptoms and response to treatment. Ultimately, the development of validated biomarkers will enable earlier and more accurate diagnosis, facilitate personalized treatment strategies, and improve the lives of individuals affected by PD.

## References

[B1] Abdi I. Y. Majbour N. K. Willemse E. A. J. van de Berg W. D. J. Mollenhauer B. Teunissen C. E. . (2021). Preanalytical stability of CSF total and oligomeric alpha-synuclein. Front. Aging Neurosci. 3:85. doi: 10.3389/fnagi.2021.638718 33762924 PMC7982944

[B2] Agliardi C. Meloni M. Guerini F. R. Zanzottera M. Bolognesi E. Baglio F. . (2021). Oligomeric α-Syn and SNARE complex proteins in peripheral extracellular vesicles of neural origin are biomarkers for Parkinson's disease. Neurobiol. Dis. 148:105185. doi: 10.1016/j.nbd.2020.105185 33217562

[B3] Al-Nimer M. S. M. Mshatat S. Abdulla H. I. (2014). Saliva α-synuclein and a high extinction coefficient protein: a novel approach in assessment biomarkers of Parkinson's disease. N. Am. J. Med. Sci. 6, 633–637. doi: 10.4103/1947-2714.147980 25599051 PMC4290052

[B4] Alves G. Lange J. Blennow K. Zetterberg H. Andreasson U. Førland M. G. . (2014). CSF Abeta42 predicts early-onset dementia in Parkinson disease. Neurology 82, 1784–1790. doi: 10.1212/WNL.0000000000000425 24748671

[B5] Anderson J.P. Walker D.E. Goldstein J. M. Banducci K. Caccavello R. J. Barbour R. . (2006). Phosphorylation of Ser-129 is the dominant pathological modification of alpha-synuclein in familial and sporadic Lewy body disease, J. Biol. Chem. 281, 29739–29752. doi: 10.1074/jbc.M600933200 16847063

[B6] Bagheri V. Khorramdelazad H. Hassanshahi G. Moghadam-Ahmadi A. Vakilian A. (2018). CXCL12 and CXCR4 in the peripheral blood of patients with Parkinson's disease. Neuroimmunomodulation 25, 201–205. doi: 10.1159/000494435 30428473

[B7] Bartl M. Dakna M. Schade S. Otte B. Wicke T. Lang E. . (2023). Blood markers of inflammation, neurodegeneration, and cardiovascular risk in early Parkinson's disease. Mov. Disord. 38, 68–81. doi: 10.1002/mds.29257 36267007

[B8] Batzu L. Rota S. Hye A. Zetterberg H. Blennow K. Aarsland D. (2022). Plasma p-tau181, neurofilament light chain and association with cognition in Parkinson's disease. NPJ Parkinsons Dis. 8:154. doi: 10.1038/s41531-022-00384-x 36371469 PMC9653432

[B9] Bellomo G. De Luca C. M. G. Paoletti F. P. Gaetani L. Moda F. Parnetti L. (2022). alpha-Synuclein seed amplification assays for diagnosing synucleinopathies: the way forward. Neurology 99, 195–205. doi: 10.1212/WNL.0000000000200878 35914941

[B10] Berlyand Y. Weintraub D. Xie S. X. Mellis I. A. Rick J. McKinstry R. C. . (2016). An Alzheimer's disease-derived biomarker signature identifies Parkinson's disease patients with dementia. PLoS One 11:e0147319. doi: 10.1371/journal.pone.0147319 26812251 PMC4727929

[B11] Bernhardt A. M. Nemati M. Boros F. A. Tóth E. A. Pál G. Molnár K. Sántha M. . (2024). α-synuclein seed amplification assays from blood-based extracellular vesicles in Parkinson's disease: an evaluation of the evidence. Mov. Disord. 39, 1269–1271. doi: 10.1002/mds.29923 38989741

[B12] Biomarkers Definitions Working Group, (2001). Biomarkers and surrogate endpoints: Preferred definitions and conceptual frame work. Clin. Pharmacol. Ther. 69, 89–95. doi: 10.1067/mcp.2001.113989 11240971

[B13] Blennow K. Biscetti L. Eusebi P Parnetti L. (2016). Cerebrospinal fluid biomarkers in Alzheimer's and Parkinson's diseases-from pathophysiology to clinical practice. Mov. Disord. 31:836–847. doi: 10.1002/mds.26656 27145480

[B14] Blommer J. Pitcher T. Mustapic M. Tran J. Hsieh C. H. Coffey C. S. . (2023). Extracellular vesicle biomarkers for cognitive impairment in Parkinson's disease. Brain 146, 195–208. doi: 10.1093/brain/awac258 35833836 PMC10060702

[B15] Boing A. N. van der Pol E. Grootemaat A. E. Coumans F. A. Sturk A. Nieuwland R. (2014). Single-step isolation of extracellular vesicles by size-exclusion chromatography. J. Extracell. Vesicles 3:23430. doi: 10.3402/jev.v3.23430 PMC4159761 25279113

[B16] Bougea A. Koros C. Stefanis L. (2019). Salivary alpha-synuclein as a biomarker for Parkinson's disease: a systematic review. J. Neural Transm. 126,1373–1382. doi: 10.1007/s00702-019-02062-4 31401695

[B17] Bridel C. van Wieringen W. N. Zetterberg H. Tijms B. M. Teunissen C. E. Alvarez-Cermeño J. C. . (2019). Diagnostic value of cerebrospinal fluid neurofilament light protein in neurology: a systematic review and meta-analysis. JAMA Neurol. 76, 1035–1048. doi: 10.1001/jamaneurol.2019.1534 31206160 PMC6580449

[B18] Brockmann K. Quadalti C. Lerche S. Rossi M. Wurster I. Baiardi S. . (2021). Association between CSF alpha-synuclein seeding activity and genetic status in Parkinson's disease and dementia with Lewy bodies. Acta Neuropathol. Commun. 9:175. doi: 10.1186/s40478-021-01276-6 34717775 PMC8556894

[B19] Caldi Gomes L. Galhoz A. Jain G. Roser A. E. Maass F. Carboni E. . (2022). Multi-omic landscape scoping of human midbrains identifies disease-relevant molecular targets and pathways in advanced-stage Parkinson's disease. Clin. Transl. Med. 12:e692. doi: 10.1002/ctm2.692 35090094 PMC8797064

[B20] Calvani R. Picca A. Landi G. Marini F. Biancolillo A. Coelho-Junior H. J. . (2020). A novel multi-marker discovery approach identifies new serum biomarkers for Parkinson's disease in older people: anEXosomesinPArkiNson disease(EXPAND)ancillary study. Geroscience 42, 1323–1334. doi: 10.1007/s11357-020-00192-2 32458283 PMC7525911

[B21] Canaslan S. Schmitz M. Maass F. Hermann P. Da Silva Correia S. . (2026). Detection of alpha-synuclein seeding activity in tear fluid in patients with Parkinson's disease. NPJ Parkinsons Dis. 12:41708653. doi: 10.1101/2025.01.16.633314 PMC12992906 41708653

[B22] Cao Z. Wu Y. Liu G. Jiang Y. Wang X. Wang Z. . (2019). Alpha-synuclein in salivary extracellular vesicles as a potential biomarker of Parkinson's disease. Neurosci. Lett. 696, 114–120. doi: 10.1016/j.neulet.2018.12.030 30579996

[B23] Cao Z. Wu Y. Liu G. Jiang Y. Wang X. Wang Z. . (2020). Differential diagnosis of multiple system atrophy-parkinsonism and Parkinson's disease using alpha synuclein and external anal sphincter electromyography. Front Neurol. 11:1043. doi: 10.3389/fneur.2020.01043 33041984 PMC7527535

[B24] Cariulo C. Martufi P. Verani M. Azzollini L. Bruni G. Weiss A. Deguire S. M. . (2019). Phospho-S129 alpha-synuclein is present in human plasma but not in cerebrospinal fluid as determined by an ultrasensitive immunoassay. Front. Neurosci. 13:889. doi: 10.3389/fnins.2019.00889 31507364 PMC6714598

[B25] Chahine L. M. Beach T. G. Brumm M. C. Adler C. H. Coffey C. S. Mosovsky S. . (2020). *In vivo* distribution of alpha-synuclein in multiple tissues and biofluids in Parkinson disease. Neurology 95, e1267–e1284. doi: 10.1212/WNL.0000000000010404 32747521 PMC7538226

[B26] Chan L. Chung C. C. Hsieh Y. C. Wu R. M. Hong C. T. (2023). Plasma extracellular vesicle tau, beta-amyloid, and alpha-synuclein and the progression of Parkinson's disease: a follow-up study. Ther. Adv. Neurol. Disord. 16:17562864221150329. doi: 10.1177/17562864221150329 36741351 PMC9896092

[B27] Chang C.-W. Yang S.-Y. Yang C.-C. Chang C.-W. Wu Y.-R. (2020). Plasma and serum alpha-synuclein as a biomarker of diagnosis in patients With Parkinson's Disease. Front. Neurol. 10:1388. doi: 10.3389/fneur.2019.01388 32038461 PMC6990107

[B28] Chatterjee K. Roy A. Banerjee R. Choudhury S. Mondal B. Halder S. . (2020). Inflammasome and alpha-synuclein in Parkinson's disease: a cross-sectional study. J. Neuroimmunol. 338:577089. doi: 10.1016/j.jneuroim.2019.577089 31704453

[B29] Che N. Ou R. Li C. Hou Y. Wei Q. Cao B. . (2024). Plasma GFAP as a prognostic biomarker of motor subtype in early Parkinson's disease. NPJ Parkinsons Dis. 10:48. doi: 10.1038/s41531-024-00664-8 38429295 PMC10907600

[B30] Chen X. Hu Y. Cao Z. Liu Q. Cheng Y. (2018). Cerebrospinal fluid inflammatory cytokine aberrations in Alzheimer's disease, parkinson's disease, and amyotrophic lateral sclerosis: a systematic review and meta-analysis. Front. Immunol. 9:2122. doi: 10.3389/fimmu.2018.02122 30283455 PMC6156158

[B31] Chiu M. J. Yang S. Y. Chen T. F. Chieh J. J. Huang T. Z. Yip P. K. . (2021). Synergistic association between plasma Abeta(1–42) and p-tau in Alzheimer's disease but not in Parkinson's disease or frontotemporal dementia. ACS Chem. Neurosci. 12,1376–1383. doi: 10.1021/acschemneuro.1c00010 PMC9278807 33825443

[B32] Chung C. C. Chan L. Chen J. H. Bamodu O. A. Chiu H. W. Hong C. T. (2021). Plasma extracellular vesicles tau and β-amyloid as biomarkers of cognitive dysfunction of Parkinson's disease. FASEB J. 35:e21895. doi: 10.1096/fj.202100787R 34478572

[B33] Church M. Chen-Plotkin A. S. Walt D. R. (2024). Measurement of alpha-synuclein as protein cargo in plasma extracellular vesicles. Proc. Natl. Acad. Sci. USA 121:e2408949121. doi: 10.1073/pnas.2408949121 39475636 PMC11551346

[B34] Clinton L. K. Blurton-Jones M. Myczek K. Trojanowski J. Q. LaFerla F. M. (2010). Synergistic interactions between abeta, tau, and alpha-synuclein: Acceleration of neuropathology and cognitive decline. J. Neurosci. 30, 7281–7289. doi: 10.1523/JNEUROSCI.0490-10.2010 20505094 PMC3308018

[B35] Compta Y. Parkkinen L. O'Sullivan S. S. Vandrovcova J. Holton J. L. Collins C. . (2011). Lewy- and Alzheimer-type pathologies in Parkinson's disease dementia: which is more important? Brain 134, 1493–1505. doi: 10.1093/brain/awr031 21596773 PMC4194668

[B36] Compta Y. Pereira J. B. Rios J. Ibarretxe-Bilbao N. Junque C. Bargallo N. . (2013). Combined dementia-risk biomarkers in Parkinson's disease: a prospective longitudinal study. Parkinsonism Relat. Disord. 19, 717–724. doi: 10.1016/j.parkreldis.2013.03.009 23643469

[B37] Concha-Marambio L. Pritzkow S. Shahnawaz M. Farris C. M. Soto C. (2023). Seed amplification assay for the detection of pathologic alpha-synuclein aggregates in cerebrospinal fluid. Nat. Protoc. 18, 1179–1196. doi: 10.1038/s41596-022-00787-3 36653527 PMC10561622

[B38] Constatidines V. C. Majbour N. K. Paraskevas G. P. Abdi I. Safieh-Garabedian B. Stefanis L. . (2021). Cerebrospinal fluid alpha-synuclein species in cognitive and movements disorders. Brain Sci. 11:119. doi: 10.3390/brainsci11010119 33477387 PMC7830324

[B39] Coughlin D. Xie S. X. Liang M. Williams A. Peterson C. Weintraub D. . (2019). Cognitive and pathological influences of tau pathology in Lewy body disorders. Ann. Neurol. 85, 259–271. doi: 10.1002/ana.25392 30549331 PMC6375484

[B40] Coughlin D. G. Irwin D. J. (2023). Fluid and biopsy based biomarkers in Parkinson's disease. Neurotherapeutics 20, 932–954. doi: 10.1007/s13311-023-01379-z 37138160 PMC10457253

[B41] Cressatti M. Juwara L. Galindez J. M. Velly A. M. Nkurunziza E. S. (2020). Salivary microR-153 and microR-223 levels as potential diagnostic biomarkers of idiopathic Parkinson's disease. Mov. Disord. 35, 468–477. doi: 10.1002/mds.27935 31800144

[B42] Daniele S. Frosini D. Pietrobono D. Petrozzi L. Lo Gerfo A. Baldacci F. . (2018). α-synuclein heterocomplexes with β-amyloid are increased in red blood cells of Parkinson's disease patients and correlate with disease severity. Front. Mol. Neurosci. 11:53. doi: 10.3389/fnmol.2018.00053 29520218 PMC5827358

[B43] de Araujo F. M. Cuenca-Bermejo L. Fernandez-Villalba E. Costa S. L. Silva V. D. A. Herrero M. T. (2021). Role of microgliosis and NLRP3 inflammasome in Parkinson's disease pathogenesis and therapy. Cell. Mol. Neurobiol. 42,1283–1300. doi: 10.1007/s10571-020-01027-6 33387119 PMC11421755

[B44] De Bartolo M. I. Vivacqua G. Belvisi D. Gaetani L. Giannini G. Mancinelli R. (2023) A combined panel of salivary biomarkers in de novo Parkinson's disease. Ann. Neurol. 93, 446–459. doi: 10.1002/ana.26550 36385395

[B45] Delenclos M. Jones D. R. McLean P. J. Uitti R. J. (2016). Biomarkers in Parkinson's disease: advances and strategies. Parkinsonism Relat. Disord. 22, S106–S110. doi: 10.1016/j.parkreldis.2015.09.048 26439946 PMC5120398

[B46] Delgado-Alvarado M. Gago B. Gorostidi A. Jimenez Urbieta H. Dacosta-Aguayo R. . (2017). Tau/alpha-synuclein ratio and inflammatory proteins in Parkinson's disease: an exploratory study. Mov. Disord. 32, 1066–1073. doi: 10.1002/mds.27001 28548309

[B47] Delgado-Alvarado M. Gago B. Navalpotro-Gomez I. Jiménez-Urbieta H. Rodriguez-Oroz M. C. (2016). Biomarkers for dementia and mild cognitive impairment in Parkinson's disease. Mov. Disord. 31, 861–881. doi: 10.1002/mds.26662 27193487

[B48] Devic I. Hwang H. Edgar J. S. Izutsu K. Presland R. Pan C. . (2011). Salivary alpha-synuclein and DJ-1: Potential biomarkers for Parkinson's disease. Brain 134:e178. doi: 10.1093/brain/awr015 21349902 PMC3122368

[B49] Dixit A. Mehta R. Singh A. K. (2019). Proteomics in human Parkinson's disease: present scenario and future directions. Cell. Mol. Neurobiol. 39, 901–915. doi: 10.1007/s10571-019-00700-9 31190159 PMC11457823

[B50] Duran R. Barrero F J. Morales B. Luna J D. Ramirez M. Vives F. (2010). Plasma alpha-synuclein in patients with Parkinson's disease with and without treatment. Mov. Disord. 25, 489–493. doi: 10.1002/mds.22928 20063406

[B51] Dutta S. Hornung S. Kruayatidee A. Maina M. B. Delanghe N. Delputte C. . (2021). Alpha-synuclein in blood exosomes immunoprecipitated using neuronal and oligodendroglial markers distinguishes Parkinson's disease from multiple system atrophy. Acta Neuropathol. 142, 495–511. doi: 10.1007/s00401-021-02324-0 33991233 PMC8357708

[B52] Eidson L. N. Kannarkat G. T. Barnum C. J. Chang J. Chung J. Caspell-Garcia C. . (2017). Candidate inflammatory biomarkers display unique relationships with alpha-synuclein and correlate with measures of disease severity in subjects with Parkinson's disease. J. Neuroinflammation 14:164. doi: 10.1186/s12974-017-0935-1 28821274 PMC5563061

[B53] El-Agnaf O. M. Salem S. A. Paleologou K. E. Curran M. D. Gibson M. J. Court J. A. . (2006). Detection of oligomeric forms of alpha-synuclein protein in human plasma as a potential biomarker for Parkinson's disease. FASEB J. 20, 419–425. doi: 10.1096/fj.03-1449com 16507759

[B54] Eusebi P. Giannandrea D. Biscetti L. Abraha I. Chiasserini D. Orso M. . (2017). Diagnostic utility of cerebrospinal fluid α-synuclein in Parkinson's disease: a systematic review and meta-analysis. Mov Disord 32, 1389–1400. doi: 10.1002/mds.27110 28880418

[B55] Fairfoul G. McGuire L. I. Pal S. Ironside J. W. Neumann J. Christie S. . (2016). Alpha-synuclein RT-QuIC in the CSF of patients with alpha-synucleinopathies. Ann. Clin. Transl. Neurol. 3, 812–818. doi: 10.1002/acn3.338 27752516 PMC5048391

[B56] Fan Z. Pan Y. T. Zhang Z. Y. Yang H. Yu S. Y. Zheng Y. Ma J. H. . (2020). Systemic activation of NLRP3 inflammasome and plasma alpha-synuclein levels are correlated with motor severity and progression in Parkinson's disease. J. Neuroinflammation 17:11. doi: 10.1186/s12974-019-1670-6 31915018 PMC6950934

[B57] Fereshtehnejad S. M. Romenets S. R. Anang J. B. Latreille V. Gagnon J. F. Postuma R. B. (2015). New clinical subtypes of Parkinson disease and their longitudinal progression: a prospective cohort comparison with other phenotypes. JAMA Neurol. 72, 863–873. doi: 10.1001/jamaneurol.2015.0703 26076039

[B58] Fereshtehnejad S. M. Zeighami Y. Dagher A. Postuma R. B. (2017). Clinical criteria for subtyping Parkinson's disease: biomarkers and longitudinal progression. Brain 140,1959–1976. doi: 10.1093/brain/awx118 28549077

[B59] Førland M. G. Tysnes O. B. Aarsland D. Maple-Grødem J. Pedersen K. F. Alves G. . (2020). The value of cerebrospinal fluid α-synuclein and the tau/α-synuclein ratio for diagnosis of neurodegenerative disorders with Lewy pathology. Eur. J. Neurol. 27, 43–50. doi: 10.1111/ene.14032 31293044

[B60] Foulds P. G. Yokota O. Thurston A. Davidson Y. Ahmed Z. Holton J. . (2012). Post mortem cerebrospinal fluid α-synuclein levels are raised in multiple system atrophy and distinguish this from the other α-synucleinopathies, Parkinson's disease and Dementia with Lewy bodies. Neurobiol. Dis. 45, 188–195. doi: 10.1016/j.nbd.2011.08.003 21856424 PMC3657198

[B61] Fraser K. B. Rawlins A. B. Clark R. G. Alcalay R. N. Standaert D. G. Liu N. . (2016). Ser(P)-1292 LRRK2 in urinary exosomes is elevated in idiopathic Parkinson's disease: P-LRRK2 predicts severity of PD. Mov. Disord. 31, 1543–1550. doi: 10.1002/mds.26686 27297049 PMC5053851

[B62] Fu Y. Jiang C. Tofaris G. K. Davis J. J. (2020). Facile impedimetric analysis of neuronal exosome markers in Parkinson's disease diagnostics. Anal. Chem. 92, 13647-13651. doi: 10.1021/acs.analchem.0c03092 32945162 PMC7584333

[B63] Ganguly U. Singh S. Pal S. Prasad S. Agrawal B. K Saini R. V. . (2021). Alpha-synuclein as a biomarker of Parkinson's disease: good, but not good enough. Front. Aging Neurosci. 13:702639. doi: 10.3389/fnagi.2021.702639 34305577 PMC8298029

[B64] Garrido A. Fairfoul G. Tolosa E. S. Martí M. J. Green A. (2019). α-synuclein RT-QuIC in cerebrospinal fluid of LRRK2-linked Parkinson's disease. Ann. Clin. Transl. Neurol. 6, 1024–1032. doi: 10.1002/acn3.772 31211166 PMC6562027

[B65] Giampa M. Amundarain M. J. Herrera M. G. Tonali N. Donero V. I. (2021). Implementing complementary approach to shape the mechanism of α-synuclein oligomerization as a model of amyloid aggregation. Molecular 27:88. doi: 10.3390/molecules27010088 35011320 PMC8747028

[B66] Goldman J. G. Andrews H. Amara A. Naito A. Alcalay R. N. Shaw L. M. . (2018). Cerebrospinal fluid, plasma, and saliva in the BioFIND study: relationships among biomarkers and Parkinson's disease features. Mov. Disord 33, 282–288. doi: 10.1002/mds.27232 29205509 PMC5836918

[B67] Grossauer A. Hemicker G. Krismer F. Peball M. Djamshidian A. Poewe W. . (2023). α-Synuclein seed amplification assays in the diagnosis of synucleinopathies using cerebrospinal fluid—a systematic review and meta-analysis. Mov. Disord Clin. Pract. 10, 737–747. doi: 10.1002/mdc3.13710 37205253 PMC10187020

[B68] Groveman B. R. Orru C. D. Hughson A. G. Raymond L. D. Zanusso G. Ghetti B. . (2018). Rapid and ultra-sensitive quantitation of disease-associated alpha-synuclein seeds in brain and cerebrospinal fluid by alphaSyn RT-QuIC. Acta Neuropathol. Commun. 6:7. doi: 10.1186/s40478-018-0508-2 29422107 PMC5806364

[B69] Guévremont D. Roy J. Cutfield N. J. Williams J. M. (2023). MicroRNAs in Parkinson's disease: a systematic review and diagnostic accuracy meta-analysis. Sci. Rep. 13:16272. doi: 10.1038/s41598-023-43096-9 37770507 PMC10539377

[B70] Gupta V. Garg R. K. Khattri S. (2016). Levels of IL-8 and TNF-alpha decrease in Parkinson's disease. Neurol. Res. 38, 98–102. doi: 10.1080/01616412.2015.1133026 27078697

[B71] Gurgone A. Cardinale V. Tangari M. M. Budicin S. Comai D. Ledda C. . (2026). Clinical validation of α-synuclein in salivary extracellular vesicles as a biomarker for Parkinson's disease: a longitudinal study. Eur. J. Neurol. 33:e70508. doi: 10.1111/ene.70508 41603154 PMC12848592

[B72] Guzowska J. Kowalski S. Schachta I. Piekuś-Słomka N. Słomka A. (2026). Connecting the dots: milestones in the history of extracellular vesicle research. Int. J. Mol. Sci. 27:2470. doi: 10.3390/ijms27052470 41828683 PMC12986264

[B73] Hall S. Janelidze S. Surova Y. Widner H. Zetterberg H. Hansson O. (2018). Cerebrospinal fluid concentrations of inflammatory markers in Parkinson's disease and atypical parkinsonian disorders. Sci. Rep. 8:13276. doi: 10.1038/s41598-018-31517-z 30185816 PMC6125576

[B74] Hall S. Surova Y. Öhrfelt A. Blennow K. Zetterberg H. Hansson O. the Swedish BioFINDE, R. (2026). Longitudinal measurements of cerebrospinal fluid biomarkers in Parkinson's disease. Mov. Disord. 31, 898–905. doi: 10.1002/mds.26578 26878815 PMC5067556

[B75] Hall S. Surova Y. Öhrfelt A. Zetterberg H. Lindqvist D. Hansson O. (2015). CSF biomarkers and clinical progression of Parkinson disease. Neurology 84, 57–63. doi: 10.1212/WNL.0000000000001098 25411441 PMC4336091

[B76] Halliday G. Hely M. Reid W. Morris J. (2008). The progression of pathology in longitudinally followed patients with Parkinson's disease. Acta Neuropathol. 115, 409–415. doi: 10.1007/s00401-008-0344-8 18231798

[B77] Hamm-Alvarez S. F. Okamoto C. T. Janga S. R. Feigenbaum D. Edman M. C. Freire D. . (2019). Oligomeric alpha-synuclein is increased in basal tears of Parkinson's patients. Biomark. Med. 13, 941–952. doi: 10.2217/bmm-2019-0167 31262201

[B78] Hansson O. Hall S. Ohrfelt A. Zetterberg H. Blennow K. Minthon L. . (2014). Levels of cerebrospinal fluid α-synuclein oligomers are increased in Parkinson's disease with dementia and dementia with Lewy bodies compared to Alzheimer's disease. Alzheimers. Res. Ther. 6:25. doi: 10.1186/alzrt255 24987465 PMC4075410

[B79] Hansson O. Janelidze S. Hall S. Magdalinou N. Lima Dias S. Constantinescu R. . (2017). Blood-based NfL: a biomarker for differential diagnosis of parkinsonian disorders. Lancet Neurol. 16, 551–560. doi: 10.1016/S1474-4422(17)30124-2 28179466 PMC5333515

[B80] Harischandra D. S. Ghaisas S. Rokad D. Zamanian M. Jin H. Anantharam V. . (2018). Environmental neurotoxicant manganese regulates exosome-mediated extracellular miRNAs in a cell culture model of Parkinson's disease. Neurotoxicology 64, 267–277. doi: 10.1016/j.neuro.2017.04.007 28450057 PMC5654692

[B81] Hatano T. Okuzumi A. Matsumoto G. Tsunemi T. Hattori N. (2024). 10.14802/jmd.24075 PMC11082597 38589016

[B82] Henderson M. X. Sengupta M. Trojanowski J. Q. Lee V. M. Y. (2019). Alzheimer's disease tau is a prominent pathology in LRRK2 Parkinson's disease. Acta Neuropathol. Commun. 7:183. doi: 10.1186/s40478-019-0836-x 31733655 PMC6858668

[B83] Herbert M. K. Aerts M. B. Beenes M. Netea-Maier R. T. Esselink R. A. Bloem B. R. . (2015). CSF neurofilament light chain is a biomarker of MSA and PSP, but not PD and DLB. Front. Neurol. 6:91. doi: 10.3389/fneur.2015.00091 25999911 PMC4419719

[B84] Hook V. Podvin S. Mosier C. Boyarko B. Seyffert L. Stringer H. . (2023). Emerging evidence for dysregulated proteome cargoes of tau-propagating extracellular vesicles driven by familial mutations of tau and presenilin. Extracell. Vesicles Circ. Nucl. Acids 4, 588–598. doi: 10.20517/evcna.2023.44 38125374 PMC10732590

[B85] Howlett D. R. Whitfield D. Johnson M. Attems J. O'brien J. T. Aarsland D. . (2015). Regional multiple pathology scores are associated with cognitive decline in lewy body dementias. Brain Pathol. 25, 401–408. doi: 10.1111/bpa.12182 25103200 PMC8029273

[B86] Hu W. T. Howell J. C. Ozturk T. Gangishetti U. Kollhoff A. L. Gangishetti U. Kollhoff A. L. Hatcher-Martin J. M. Tsvetkova D. Z. . (2019). CSF cytokines in aging, multiple sclerosis, and dementia. Front. Immunol. 10:480. doi: 10.3389/fimmu.2019.00480 30930904 PMC6428695

[B87] Hu X. Yang Y. Gong D. (2017). Changes of cerebrospinal fluid Aβ42, t-tau, and p-tau in Parkinson's disease patients with cognitive impairment relative to those with normal cognition: a meta-analysis. Neurol. Sci. 38, 1953–1961. doi: 10.1007/s10072-017-3088-1 28808876

[B88] Hu Y. Yu S. Y. Zuo L. J. Cao C. J. Wang F. Chen Z. J. . (2015). Parkinson disease with REM sleep behavior disorder: features, alpha-synuclein, and inflammation. Neurology 84, 888–894. doi: 10.1212/WNL.0000000000001308 25663225

[B89] Irwin D. J. Coughlin D. Nevler N. Akhtar R. S. McMillan C. T. Lee E. B. . (2017). Antemortem CSF tau and a beta biomarkers are predictive of postmortem alzheimer's disease pathology in autopsy confirmed lewy body disease. Ann. Neurol. 81, S56–S56.

[B90] Irwin D. J. Fedler J. Coffey C. S. Caspell-Garcia C. Kang J. H. Simuni T. . (2020). Evolution of Alzheimer's disease cerebrospinal fluid biomarkers in early Parkinson's disease. Ann. Neurol. 88, 574–587. doi: 10.1002/ana.25811 32542885 PMC7497251

[B91] Irwin D. J. Lee V. M.-Y. Trojanowski J. Q. (2013). Parkinson's disease dementia: convergence of α-synuclein, tau and amyloid-β pathologies. Nat. Rev. Neurosci. 14, 626–636. doi: 10.1038/nrn3549 23900411 PMC4017235

[B92] Ishiguro Y. Tsunemi T. Shimada T. Saito K. Furukawa Y. Hatano T. . (2024). Extracellular vesicles contain filamentous alpha-synuclein and facilitate the propagation of Parkinson's pathology. Biochem. Biophys. Res. Commun. 703:149620. doi: 10.1016/j.bbrc.2024.149620 38359614

[B93] Ishiki A. Kamada M. Kawamura Y. Terao C. Shimoda F. Tomita N. . (2016). Glial fibrillary acidic protein in the cerebrospinal fluid of alzheimer's disease, dementia with lewy bodies, and frontotemporal lobar degeneration. J. Neurochem. 136, 258–261. doi: 10.1111/jnc.13399 26485083

[B94] Iwaoka K. Otsuka C. Maeda T. Yamahara K. Kato K. Takahashi M. Yamada K. (2020). Impaired metabolism of kynurenine and its metabolites in CSF of Parkinson's disease. Neurosci. Lett. 714:134576. doi: 10.1016/j.neulet.2019.134576 31654722

[B95] Jellinger K A. (2008). Neuropathological aspects of Alzheimer disease, Parkinson disease and frontotemporal dementia. Neurodegener. Dis. 5, 118–121. doi: 10.1159/000113679 18322367

[B96] Jellinger K. A. Seppi K. Wenning G. K. Poewe W. (2002). Impact of coexistent Alzheimer pathology on the natural history of Parkinson's disease. J. Neural Transm. 109, 329–339. doi: 10.1007/s007020200027 11956955

[B97] Jiang C. Hopfner F. Katsikoudi A. Hein R. Catli C. Evetts S. . (2020). Serum neuronal exosomes predict and differentiate Parkinson's disease from atypical parkinsonism. J. Neurol. Neurosurg. Psychiatr. 91, 720–729. doi: 10.1136/jnnp-2019-322588 32273329 PMC7361010

[B98] Jiang Y. Chen J. Sun Y. Li F. Wei L. Sun W. Sun W. . (2021). Profiling of differentially expressed microRNAs in Saliva of Parkinson's disease patients. Front. Neurol. 12:738530. 34899562 10.3389/fneur.2021.738530 PMC8660675

[B99] Jin H. Gu H.Y. Mao C.J. Chen J. Liu C.F. (2020). Association of inflammatory factors and aging in Parkinson's disease. Neurosci. Lett. 736:135259. doi: 10.3389/fneur.2021.738530 32682845

[B100] Kaerst L. Kuhlmann A. Wedekind D. Stoeck K. Lange P. Zerr I. (2013). Using cerebrospinal fluid marker profiles in clinical diagnosis of dementia with lewy bodies, Parkinson's Disease, and Alzheimer's Disease. J Alzheimer Dis 38, 63–73. doi: 10.3233/JAD-130995 23948928

[B101] Kang J.-H. Mollenhauer B. Coffey C. S. Toledo J. B. Weintraub D. Galasko D. R. . (2016). CSF biomarkers associated with disease heterogeneity in early Parkinson's disease: the Parkinson's progression markers initiative study. Acta Neuropathol. 131, 935–949. doi: 10.1007/s00401-016-1552-2 27021906 PMC5031365

[B102] Kang J. H. Irwin D. J. Chen-Plotkin A. S. Siderowf A. Caspell C. Coffey C. S. . (2013). Association of cerebrospinal fluid beta-amyloid 1–42, T-tau, P-tau181, and alpha-synuclein levels with clinical features of drug-naive patients with early Parkinson disease. JAMA Neurol. 70, 1277–1287. doi: 10.1001/jamaneurol.2013.3861 23979011 PMC4034348

[B103] Kang U. J. Boehme A. K. Fairfoul G. Shahnawaz M. Ma T. C. Hutten S. J. . (2019). Comparative study of cerebrospinal fluid alpha-synuclein seeding aggregation assays for diagnosis of Parkinson's disease. Mov. Disord. 34, 536–544. doi: 10.1002/mds.27646 30840785 PMC6519150

[B104] Karpenko M. N. Vasilishina A. A. Gromova E. A. Muruzheva Z. M. Miliukhina I. V. Bernadotte A. (2018). Interleukin-1beta, interleukin-1 receptor antagonist, interleukin-6, interleukin-10, and tumor necrosis factor-alpha levels in CSF and serum in relation to the clinical diversity of Parkinson's disease. Cell. Immunol. 327, 77–82. doi: 10.1016/j.cellimm.2018.02.011 29478949

[B105] Keighron C. N. Avazzadeh S. Goljanek-Whysall K. McDonagh B. Howard L. Ritter T. . (2023). Extracellular Vesicles, Cell-Penetrating Peptides and miRNAs as Future Novel Therapeutic Interventions for Parkinson's and Alzheimer's Disease. Biomedicines 11:728. doi: 10.3390/biomedicines11030728 36979707 PMC10045119

[B106] Khalil M. Teunissen C. E. Lehmann S. Otto M. Piehl F. Sormani M. P. . (2024). Neurofilaments as biomarkers in neurological disorders: toward clinical application. Nat. Rev. Neurol. 20, 269–287. doi: 10.1038/s41582-024-00955-x 38609644

[B107] Kharel S. Ojha R. Bist A. Joshi S. P. Rauniyar R. Yadav J. K. (2022). Salivary alpha-synuclein as a potential fluid biomarker in Parkinson's disease: a systematic review and meta-analysis. Aging Med. 5, 53–62. doi: 10.1002/agm2.12192 35309157 PMC8917264

[B108] Kim K. Y. Shin K. Y. Chang K. A. (2024). Potential exosome biomarkers for Parkinson's disease diagnosis: a systematic review and meta-analysis. Int. J. Mol. Sci. 25:5307. doi: 10.3390/ijms25105307 38791346 PMC11121363

[B109] Kim R. Kim H. J. Kim A. Jang M. Kim Y. Yoo D. . (2018). Peripheral blood inflammatory markers in early Parkinson's disease. J. Clin. Neurosci. 58, 30–33. doi: 10.1016/j.jocn.2018.10.079 30454693

[B110] Kluge A. Schaeffer E. Bunk J. Sommerauer M. R?ttgen S. Schulte C. . (2024). Detecting misfolded a-synuclein in blood years before the diagnosis of Parkinson's disease. Mov. Disord. 39, 1289–1299. doi: 10.1002/mds.29766 38651526

[B111] Koga S. Sekiya H. Kondru N. Ross O. A. Dickson D. W. (2021). Neuropathology and molecular diagnosis of synucleinopathies. Mol Neurodegener 16:83. doi: 10.1186/s13024-021-00501-z 34922583 PMC8684287

[B112] Kouchaki E. Kakhaki R. D. Tamtaji O. R. Dadgostar E. Behnam M. Nikoueinejad H. (2018). Increased serum levels of TNF-alpha and decreased serum levels of IL-27 in patients with Parkinson disease and their correlation with disease severity. Clin. Neurol. Neurosurg. 166, 76–79. doi: 10.1016/j.clineuro.2018.01.022 29408778

[B113] Kouli A. Williams-Gray C. H. (2020). Timing is everything: the *T*-cell response to alpha-synuclein is maximal in early Parkinson's. Mov. Disord. 35:1137. doi: 10.1002/mds.28122 32557803

[B114] Kurata T. Kawarabayashi T. Murakami T. Nishimura M. Morimoto N. Kawai H. . (2007). Enhanced accumulation of phosphorylated alpha-synuclein in double transgenic mice expressing mutant beta-amyloid precursor protein and presenilin-1. J. Neurosci. Res. 85, 2246–2252. doi: 10.1002/jnr.21352 17526016

[B115] Latifkar A. Hur Y. H. Sanchez J. C. Cerione R. A. Antonyak M. A. (2019). New insights into extracellular vesicle biogenesis and function. J. Cell Sci. 132:jcs222406. doi: 10.1242/jcs.222406 31263077 PMC6633391

[B116] Leaver K. Poston K. L. (2015). Do CSF biomarkers predict progression to cognitive impairment in Parkinson's disease patients? A systematic review. Neuropsychol. Rev. 25, 411–423. doi: 10.1007/s11065-015-9307-8 26626621 PMC5152566

[B117] Lee A. Gilbert R. M. (2016). Epidemiology of Parkinson's disease. Neurol. Clin. 34, 955–966. doi: 10.1016/j.ncl.2016.06.012 27720003

[B118] Lerche S. Zimmermann M. WursterI Roeben B. Fries F.L. Deuschle C. . (2022). CSF and serum levels of inflammatory markers in PD: Sparse correlation, sex differences and association with neurodegenerative biomarkers. Front. Neurol. 13:834580. 35280273 10.3389/fneur.2022.834580 PMC8914943

[B119] Li P. Y. Xu P. Y. Li J. (2021). Editorial: biomarkers and pathogenesis of alpha Synuclein in Parkinson's disease. Front. Aging Neurosci. 13:776873. doi: 10.3389/fnagi.2021.776873 34675800 PMC8523817

[B120] Li X. Yang W. Li X. Li X. Chen Z. Wang C. . (2019). Levels of oligomeric alpha-synuclein in red blood cells are elevated in patients with Parkinson's disease and affected by brain alpha-synuclein expression. Int. J. Clin. Exp. Med. 12, 10470–10477.

[B121] Li X. Y. Li W. Li X. Li X. R. Sun L. Yang W. . (2021). Alterations of erythrocytic phosphorylated alpha-synuclein in different subtypes and stages of Parkinson's disease. Front. Aging Neurosci. 13:623977. doi: 10.3389/fnagi.2021.623977 34658833 PMC8511781

[B122] Li Y. Cao Y. Liu W. Chen F. Zhang H. Zhou H. . (2024). Candidate biomarkers of EV- microRNA in detecting REM sleep behavior disorder and Parkinson's disease. NPJ Parkinsons Dis. 10:18. doi: 10.1038/s41531-023-00628-4 38200052 PMC10781790

[B123] Li Y. Yang Y. Zhao A. Luo N. Niu M. Kang W. . (2022). Parkinson's disease peripheral immune biomarker profile: a multicentre, cross-sectional and longitudinal study. J. Neuroinflammation 19:116. doi: 10.1186/s12974-022-02481-3 35610646 PMC9131564

[B124] Lian T. H. Guo P. Zuo L. J. Hu Y. Yu S. Y. Yu Q. J. . (2019). Tremor-dominant in Parkinson disease: the relevance to iron metabolism and inflammation. Front. Neurosci. 13:255. doi: 10.3389/fnins.2019.00255 30971879 PMC6445850

[B125] Liddelow S. A. Barres B. A. (2017). Reactive astrocytes: production, function, and therapeutic potential. Immunity 46, 957–967. doi: 10.1016/j.immuni.2017.06.006 28636962

[B126] Lin C. H. Li C. H. Yang K. C. Lin F. J. Wu C. C. Chieh J. J. . (2019a). Blood NfL: a biomarker for disease severity and progression in Parkinson disease. Neurology 93, e1104–e1111. doi: 10.1212/WNL.0000000000008088 31420461

[B127] Lin C. H. Liu H. C. Yang S. Y. Yang K. C. Wu C. C. Chiu M. J. (2019b). Plasma pS129-α-Synuclein is a surrogate biofluid marker of motor severity and progression in Parkinson's disease. J. Clin. Med. 8:1601. doi: 10.3390/jcm8101601 31623323 PMC6832465

[B128] Lin C. H. Yang S. Y. Horng H. E. Yang C. C. Chieh J. J. Chen H. H. . (2017). Plasma a-synuclein predicts cognitive decline in Parkinson's disease. J. Neurol. Neurosurg. Psychiatry 88, 818–824. doi: 10.1136/jnnp-2016-314857 28550072 PMC5629933

[B129] Lin J. Ou R. Li C. Hou Y. Wei Q. Cao B. . (2023). Plasma glial fibrillary acidic protein as a biomarker of disease progression in Parkinson's disease: a prospective cohort study. BMC Med. 21:420. doi: 10.1186/s12916-023-03120-1 37932720 PMC10626747

[B130] Lin J. Zhang M. Yu C Q. Xue M. Hu P P. (2025). Early diagnosis of Parkinson's disease: biomarker study. Front. Aging Neurosci. 17:1495769. doi: 10.3389/fnagi.2025.1495769 40416739 PMC12098601

[B131] Lindestam Arlehamn C. S. Dhanwani R. Pham J. Kuan R. Frazier A. . (2020). Alpha Synuclein-specific T cell reactivity is associated with preclinical and early Parkinson's disease. Nat. Commun. 11:1875. doi: 10.1038/s41467-020-15626-w 32313102 PMC7171193

[B132] Lindqvist D. Hall S. Surova Y. Nielsen H. M. Janelidze S. Brundin L. . (2013). Cerebrospinal fluid inflammatory markers in Parkinson's disease–associations with depression, fatigue, and cognitive impairment. Brain. Behav. Immun. 33, 183–189. 23911592 10.1016/j.bbi.2013.07.007

[B133] Liu T. Zuo H. Ma D. Song D. Zhao Y. Cheng O. (2023). Cerebrospinal fluid GFAP is a predictive biomarker for conversion to dementia among *de novo* Parkinson's disease patients. J. Neuroinflammation 20:167. doi: 10.1186/s12974-023-02843-5 37475029 PMC10357612

[B134] Lotankar S. Prabhavalkar K. S. Bhatt L. K. (2017). Biomarkers for Parkinson's disease: recent advancement. Neurosci. Bull. 33, 585–597. doi: 10.1007/s12264-017-0183-5 28936761 PMC5636742

[B135] Luo H. Yu X. Li P. Hu J. Li W. Li X. . (2024). Different neurotoxicity and seeding activity between α-synuclein oligomers formed in plasma of patients with Parkinson's disease and multiple system atrophy. Neuroscience 557, 1–11. doi: 10.1016/j.neuroscience.2024.08.006 39127345

[B136] Ma J. Tang Z. Wu Y. Liu Y. Zhang H. Wang X. . (2025). Differences in blood and cerebrospinal fluid between Parkinson's disease and related disorders. Cell. Mol. Neurobiol. 45, 1–15. doi: 10.1007/s10571-024-01523-z 39729132 PMC11680620

[B137] Ma L. Z. Zhang C. Wang H. Ma Y. H. Shen X. N. Wang J. . (2021). Serum neurofilament dynamics predicts cognitive progression in de novo Parkinson's disease. J. Parkinsons Dis. 11, 1117–1127. doi: 10.3233/JPD-212535 33935105

[B138] Ma Y. Farris C. M. Weber S. Wang Z. Soto C. Kang U. J. . (2024). Sensitivity and specificity of a seed amplification assay for diagnosis of multiple system atrophy: a multicentre cohort study. Lancet Neurol. 23, 1225–1237. doi: 10.1016/S1474-4422(24)00395-8 39577923 PMC12288831

[B139] Maass F. Rikker S. Dambeck V. Schäfer J. Kwiecinski H. Schlüter H. . (2020). Increased alpha-synuclein tear fluid levels in patients with Parkinson's disease. Sci. Rep. 10:8507. doi: 10.1038/s41598-020-65503-1 32444780 PMC7244583

[B140] Magdalinou N. K. Noyce A. J. Pinto R. Lindstrom E. Holmen-Larsson J. . (2017). Identification of candidate cerebrospinal fluid biomarkers in parkinsonism using quantitative proteomics. Parkinsonism. Relat. Disord. 37, 65–71. doi: 10.1016/j.parkreldis.2017.01.016 28214264

[B141] Magdalinou N. K. Paterson R. W. Schott J. M. Fox N. C. Mummery C. Blennow K. . (2015). A panel of nine cerebrospinal fluid biomarkers may identify patients with atypical parkinsonian syndromes. J. Neurol. Neurosurg. Psychiatr. 86, 1240–1247. doi: 10.1136/jnnp-2014-309562 25589779 PMC4564944

[B142] Maiese K. (2022). Biomarkers for Parkinson's disease and neurodegenerative disorders: a role for non-coding RNAs. Curr. Neurovasc. Res. 19, 127–130. doi: 10.2174/1567202619666220602125806 35657043

[B143] Majbour N. Msc N. N. V. Eusebi P. Chiasserini D. Ardah M. Varghese S. . (2016). Longitudinal changes in CSF alpha-synuclein species reflect Parkinson's disease progression. Mov. Disord. 31, 1535–1542. doi: 10.1002/mds.26754 27548849

[B144] Majbour N. K. Aasly J. O. Husta E. Thomas M. A. Vaikath N. N. Eliezer D. . (2020). CSF total and oligomeric α-Synuclein along with TNF-αas risk biomarkers for Parkinson's disease: a study in LRRK2 mutation carriers. Transl. Neurodegener. 9:15. doi: 10.1186/s40035-020-00192-4 32375873 PMC7201744

[B145] Majbour N. K. Abdi I. Y. Dakna M. Vaikath N. N. Ardah M. T. Van Dijk K. D. . (2021). Cerebrospinal α-synuclein oligomers reflect disease motor severity in DeNoPa longitudinal cohort. Mov. Disord. 36, 2048–2056. doi: 10.1002/mds.28611 33978256

[B146] Majbour N. K. Vaikath N. N. van Dijk K. D. Ardah M. T. Vesterager L. B. . (2016). Oligomeric and phosphorylated alpha-synuclein as potential CSF biomarkers for Parkinson's disease. Mol. Neurodegener. 11:7. doi: 10.1186/s13024-016-0072-9 26782965 PMC4717559

[B147] Mao C. Wang Y. Cui S. Xu X. (2023). Association of serum neurofilament light chain and glial fibrillary acidic protein levels with cognitive decline in Parkinson's disease. Brain Res. 1805:148271. doi: 10.1016/j.brainres.2023.148271 36754139

[B148] Margis R. Margis R. Rieder C. R. (2011). Identification of blood microRNAs associated to Parkinson disease. J. Biotechnol. 152, 96–101. doi: 10.1016/j.jbiotec.2011.01.023 21295623

[B149] Martin-Ruiz C. Williams-Gray C. H. Yarnall A. J. Boucher J. J. Lawson R. A. Fiandaca M. . (2020). Senescence and inflammatory markers for predicting clinical progression in Parkinson's disease: the ICICLEPD study. J. Parkinsons. Dis. 10, 193–206. doi: 10.3233/JPD-191724 31868677 PMC7029330

[B150] Meloni M. Agliardi C. Guerini F. R. Zanzottera M. Bolognesi M. Bruni A. C. (2023). Oligomeric alpha- synuclein and tau aggregates in NDEVs differentiate Parkinson's disease from atypical parkinsonisms. Neurobiol. Dis. 176:105947. doi: 10.1016/j.nbd.2022.105947 36481435

[B151] Miliukhina I. V. Usenko T. S. Senkevich K. A. Nikolaev M. A. Timofeeva A. A. Kolesnikova T. O. . (2020). Plasma cytokines profile in patients with Parkinson's disease associated with mutations in GBA gene. Bull. Exp. Biol. Med. 168, 423–426. doi: 10.1007/s10517-020-04723-x 32146630

[B152] Miyamoto K. S Saiki, H Matsumoto Sato, S. Imamichi Y. Yamamoto M. . (2022). Systemic metabolic alteration dependent on the thyroid- liver axis in early PD. Ann. Neurol. 93, 21. doi: 10.1002/ana.26510 36128871 PMC10092289

[B153] Mollenhauer B. Caspell-Garcia C. J. Coffey C. S. Taylor P. Shaw L. M. Galasko D. R. . (2017). Longitudinal CSF biomarkers in patients with early Parkinson disease and healthy controls. Neurology 89, 1959–1969. doi: 10.1212/WNL.0000000000004609 29030452 PMC5679418

[B154] Mollenhauer B. Locascio J. J. Schulz-Schaeffer W. Sixel-Döring F. Trenkwalder C. Schlossmacher M. G. (2011). Alpha-synuclein and tau concentrations in cerebrospinal fluid of patients presenting with parkinsonism: a cohort study. Lancet. Neurol. 10, 230–240. doi: 10.1016/S1474-4422(11)70014-X 21317042

[B155] Mollenhauer B. Ms C. J. C. Coffey C. S. Taylor P. Singleton A. Galasko D. . (2019). Longitudinal analyses of cerebrospinal fluid α-Synuclein in prodromal and early Parkinson's disease. Mov. Disord. 34, 1354–1364. doi: 10.1002/mds.27806 31361367 PMC7098385

[B156] Mollenhauer B. Zimmermann J. Sixel-Doring F. Focke N. K. Wicke T. DeNoPa Study Group, (2019). Baseline predictors for progression 4 years after Parkinson's disease diagnosis in the *De Novo* Parkinson Cohort (DeNoPa). Mov. Disord. 34, 67–77. doi: 10.1002/mds.27492 30468694

[B157] Montine T. J. Shi M. Quinn J. F. Peskind E. R. Craft S. Ginghina C. . (2010). CSF Aβ42 and tau in Parkinson's disease with cognitive impairment. Mov. Disord. 25, 2682–2685. doi: 10.1002/mds.23287 20818673 PMC2978754

[B158] Nayak A. Salt G. Verma S. K. Kishore U. (2015). Proteomics approach to identify biomarkers in neurodegenerative diseases. Int. Rev. Neurobiol. 121, 59–86. doi: 10.1016/bs.irn.2015.05.003 26315762

[B159] Nie C. Y. Sun H. Zhen Zhang L. Wang Y. Li H. . (2020). Differential expression of plasma exo- miRNA in neurodegenerative diseases by next- generation sequencing. Front. Neurosci. 14:438. doi: 10.3389/fnins.2020.00438 32457573 PMC7227778

[B160] Niemman L. Lezius S. Maceski A. Leppert D. Englisch C. Schwedhelm E. . (2021). Serum neurofilament is associated with motor function, cognitive decline and subclinical cardiac damage in advanced Parkinson's disease (MARK-PD). Parkinsonism Relat. Disord. 90, 44–48. 34352610 10.1016/j.parkreldis.2021.07.028

[B161] Niu M. Li Y. Li G. Chen W. Zhang J. Wang X. . (2020). A longitudinal study on alpha-synuclein in plasma neuronal exosomes as a biomarker for Parkinson's disease development and progression. Eur. J. Neurol. 27, 967–974. doi: 10.1111/ene.14208 32150777

[B162] Norman M. Ter-Ovanesyan D. Trieu W. Lazarovits R. Kowal J. Lee J. H. . (2021). L1CAM is not associated with extracellular vesicles in human cerebrospinal fluid or plasma. Nat. Methods 18, 631–634. doi: 10.1038/s41592-021-01174-8 34092791 PMC9075416

[B163] Oeckl P. Halbgebauer S. Anderl-Straub S. Oeckl P. Halbgebauer S. Anderl-Straub S. (2019). Glial fibrillary acidic protein in serum is increased in Alzheimer's disease and correlates with cognitive impairment. J. Alzheimers. Dis. 67, 481–488. doi: 10.3233/JAD-180325 30594925

[B164] Ohmichi T. Mitsuhashi M. Tatebe H. Kasai T. El-Agnaf O. M. A. Tokuda T. (2019). Quantification of brain-derived extracellular vesicles in plasma as a biomarker to diagnose Parkinson's and related diseases. Parkinsonism. Relat. Dis. 61, 82–87. doi: 10.1016/j.parkreldis.2018.11.021 30502924

[B165] Okuzumi A. Hatano T. Matsumoto G. Nojiri S. Komatsu M. Suzuki M. . (2023). Propagative alpha-synuclein seeds as serum biomarkers for synucleinopathies. Nat. Med. 29, 1448–1455. doi: 10.1038/s41591-023-02358-9 37248302 PMC10287557

[B166] Olsson B. Constantinescu R. Holmberg B. Andreasen N. Blennow K. Zetterberg H. (2013). The glial marker YKL-40 is decreased in synucleinopathies. Mov. Disord. 28, 1882–1885. doi: 10.1002/mds.25589 23847144

[B167] Oosterveld L. P. Verberk I. M. W. Majbour N. K. El-Agnaf O. M. Weinstein H. C. . (2020). CSF or serum neurofilament light added to alpha-Synuclein panel discriminates Parkinson's from controls. Mov Disord 35, 288–295. doi: 10.1002/mds.27897 31737952 PMC7027879

[B168] Oueslati A. (2016). Implication of alpha-synuclein phosphorylation at S129 in synucleinopathies: what have we learned in the past decade? J. Parkinsons. Dis. 6, 39–51. doi: 10.3233/JPD-160779 27003784 PMC4927808

[B169] Oueslati A. Fournier M. Lashuel H. A. (2010). Role of post-translational modifications in modulating the structure, function, and toxicity of alpha-synuclein: implications for Parkinson's disease pathogenesis and therapies. Prog. Brain Res. 183, 115–145. doi: 10.1016/S0079-6123(10)83007-9 20696318

[B170] Pagonabarraga J. Perez-Gonzalez R. Bejr-Kasem H. Gironell A. García-Sanchez C. Campdelacreu J. . (2022). Dissociable contribution of plasma NfL and p-tau181 to cognitive impairment in Parkinson's disease. Parkinsonism. Relat. Disord. 105, 132–138. doi: 10.1016/j.parkreldis.2022.05.020 35752549

[B171] Paoletti F. P. Gaetani L. Parnetti L. (2020). The challenge of disease-modifying therapies in Parkinson's disease: role of CSF Biomarkers. Biomolecules 10:335. doi: 10.3390/biom10020335 32092971 PMC7072459

[B172] Papagiannakis N. Koros C. Stamelou M. Rizos A. Xilouri M. Stefanis L. (2018). Alpha-synuclein dimerization in erythrocytes of patients with genetic and non- genetic forms of Parkinson's disease. Neurosci. Lett. 672, 145–149. doi: 10.1016/j.neulet.2017.11.012 29129675

[B173] Park M. J. Cheon S. M. Bae H. R. Kim S. H. Kim J. W. (2011). Elevated levels of alpha-synuclein oligomer in the cerebrospinal fluid of drug-naive patients with Parkinson's disease. J. Clin. Neurol. 7, 215–222. doi: 10.3988/jcn.2011.7.4.215 22259618 PMC3259496

[B174] Parnetti L. Farotti L. Eusebi P. Chiasserini D. De Carlo C. Giannandrea D. . (2014). Differential role of CSF alpha-synuclein species, tau, and Abeta42 in Parkinson's disease. Front. Aging Neurosci. 6:53. doi: 10.3389/fnagi.2014.00053 24744728 PMC3978246

[B175] Parnetti L. Gaetani L. Eusebi P. Paciotti S. Parnetti P. Chiasserini D. . (2019). CSF and blood biomarkers for Parkinson's disease. Lancet. Neurol. 18, 573–586. doi: 10.1016/S1474-4422(19)30024-9 30981640

[B176] Pedersen C. C. Groden J. M. Lange J. (2025). A systematic review of biofluid phosphorylated α-synuclein in Parkinson's disease. Parkinsonism Relat. Disord. 132:107240. doi: 10.1016/j.parkreldis.2024.107240 39721932

[B177] Perner C. Perner F. Gaur N. Zimmermann S. Witte O. W. Storch A. (2019). Plasma VCAM1 levels correlate with disease severity in Parkinson's disease. J. Neuroinflammation 16:94. doi: 10.1186/s12974-019-1482-8 31068198 PMC6507178

[B178] Pinotsi D. Buell A. K. Dobson C. M. Kaminski Schierle G.S. Kaminski C. F. (2013). A label-free, quantitative assay of amyloid fibril growth based on intrinsic fluorescence. ChemBioChem 14, 846–850. doi: 10.1002/cbic.201300103 23592254 PMC3790954

[B179] Poewe W. Seppi K. Tanner C. M. Halliday G. M. Brundin P. Volkmann J. . (2017). Parkinson disease. Nat. Rev. Dis. Prim. 3:17013. doi: 10.1038/nrdp.2017.13 28332488

[B180] Poggiolini I. Gupta V. Lawton M. Rossi M. Fazio P. Picillo M. . (2022). Diagnostic value of cerebrospinal fluid alpha-synuclein seed quantification in synucleinopathies. Brain 145, 584–595. doi: 10.1093/brain/awab431 34894214 PMC9014737

[B181] Postuma R. B. Berg D. Stern M. Poewe W. Olanow C. W. Oertel W. H. . (2015). MDS clinical diagnostic criteria for Parkinson's disease. Mov. Disord. 30, 1591–1601. doi: 10.1002/mds.26424 26474316

[B182] Pötter-Nerger M. Dutke J. Lezius S. Buhmann C. Schulz R. Gerloff C. Kuhle J. Choe C. U. (2022). Serum neurofilament light chain and postural instability/gait difficulty (PIGD) subtypes of Parkinson's disease in the MARK-PD study. J Neural Transm (Vienna). 129, 295–300. doi: 10.1007/s00702-022-02464-x 35072765 PMC8930951

[B183] Poulopoulos M. Levy O. A. Alcalay R. N. (2012). The neuropathology of genetic Parkinson's disease. Mov. Disord. 27, 831–842. doi: 10.1002/mds.24962 22451330 PMC3383342

[B184] Qiu X. Xiao Y. Wu J. Gan L. Huang Y. Wang J. (2019). C-reactive protein and risk of Parkinson's disease: a systematic review and meta-analysis. Front. Neurol. 10:384. doi: 10.3389/fneur.2019.00384 31057478 PMC6478798

[B185] Quek C. Hill A. F. (2017). The role of extracellular vesicles in neurodegenerative diseases. Biochem. Biophys. Res. Commun. 483, 1178–1186. doi: 10.1016/j.bbrc.2016.09.090 27659705

[B186] Rani K. Mukherjee R. Singh E. Kumar S. Sharma V. Vishwakarma P. . (2019). Neuronal exosomes in saliva of Parkinson's disease patients: a pilot study. Parkinsonism Relat. Disord. 67, 21–23. doi: 10.1016/j.parkreldis.2019.09.008 31621600

[B187] Ransohoff R. M. (2016). How neuroinflammation contributes to neurodegeneration. Science . 353, 777–783. doi: 10.1126/science.aag2590 27540165

[B188] Rastogi S. Rani K. Rai S. Singh R. Bharti P. S. . (2023). Fluorescence-tagged salivary small extracellular vesicles as a nanotool in early diagnosis of Parkinson's disease. BMC Med. 21:335. doi: 10.1186/s12916-023-03031-1 37667227 PMC10478478

[B189] Rizzo G. Copetti M. Arcuti S. Martino D. Logroscino G. (2016). Accuracy of clinical diagnosis of Parkinson's disease: a systematic review and meta-analysis. Neurology 86, 566–576. doi: 10.1212/WNL.0000000000002350 26764028

[B190] Rocha N. P. Assis F. Scalzo P. L. Vieira E. L. M. Barbosa I. G. Teixeira A. L. . (2018). Reduced activated T lymphocytes (CD4+CD25+) and plasma levels of cytokines in Parkinson's disease. Mol. Neurobiol. 55, 1488–1497. doi: 10.1007/s12035-017-0404-y 28176275

[B191] Rochester L. Galna B. Lord S. Yarnall A. Morris R. Duncan G. . (2017). Decrease in Aβ42 predicts dopa-resistant gait progression in early Parkinson disease. Neurology 88, 1501–1511. doi: 10.1212/WNL.0000000000003840 28330963 PMC5395075

[B192] Rossi M. Candelise N. Baiardi S. Mammana A. Capellari S. Parchi P. (2020). Ultrasensitive RT-QuIC assay with high sensitivity and specificity for Lewy body- associated synucleinopathies. Acta Neuropathol. 140, 49–62. doi: 10.1007/s00401-020-02160-8 32342188 PMC7299922

[B193] Russo M. J. Orru C. D. Concha-Marambio L. Llanos C. Cortes J. M. Smirnov I. . (2021). High diagnostic performance of independent alpha-synuclein seed amplification assays for detection of early Parkinson's disease. Acta Neuropathol. Commun. 9:179. doi: 10.1186/s40478-021-01292-6 34742348 PMC8572469

[B194] Sabaei M. Rahimian S. Haj Mohamad Ebrahim Ketabforoush A. Rasoolijazi H. Zamani B. Rezaei M. . (2023). Salivary levels of disease-related biomarkers in the early stages of Parkinson's and Alzheimer's disease: a cross-sectional study. IBRO Neurosci. Rep. 14, 285–292. doi: 10.1016/j.ibneur.2023.03.004 36942319 PMC10023984

[B195] Sabbagh M. N. Adler C. H. Lahti T. J. Connor D. J. Vedders L. Peterson L. K. . (2009). Parkinson disease with dementia: comparing patients with and without Alzheimer pathology. Alzheimer Dis. Assoc. Disord. 23, 295–297. doi: 10.1097/WAD.0b013e31819c5ef4 19812474 PMC2760034

[B196] Sanjari Moghaddam H. Valitabar Z. Ashraf-Ganjouei A. Mojtahed Zadeh M. Ghazi Sherbaf F. Aarabi M. H. (2018). Cerebrospinal fluid C-reactive protein in Parkinson's disease: associations with motor and non-motor symptoms. Neuromolecular. Med. 20, 376–385. doi: 10.1007/s12017-018-8499-5 29980980

[B197] Santaella A. Kuiperij H. B. van Rumund A. Esselink R. A. J. van Gool A. J. Verbeek M. M. (2020). Inflammation biomarker discovery in Parkinson's disease and atypical parkinsonisms. BMC Neurol. 20:26. doi: 10.1186/s12883-020-1608-8 31952511 PMC6967088

[B198] Schmid A. W. Fauvet B. Moniatte M. Lashuel H. A. (2013). Alpha-synuclein post-translational modifications as potential biomarkers for Parkinson disease and other synucleinopathies. Mol. Cell. Proteomics 12, 3543–3558. doi: 10.1074/mcp.R113.032730 23966418 PMC3861707

[B199] Schroder J. B. Pawlowski M. Meyer Z. Horste G. Gross C. C. Leypoldt F. (2018). Immune cell activation in the cerebrospinal fluid of patients with Parkinson's disease. Front. Neurol. 9:1081. doi: 10.3389/fneur.2018.01081 30619041 PMC6305582

[B200] Schulz I. Kruse N. Gera R. G. Kremer T. Cedarbaum J. Berg D. (2021). Systematic assessment of 10 biomarker candidates focusing on α-synuclein-related disorders. Mov. Disord. 36, 2874–2887. doi: 10.1002/mds.28738 34363416

[B201] Shaheen H. Sobhy S. El Mously S. Abuomira M. Mansour M. (2020). Salivary alpha-synuclein (total and oligomeric form): potential biomarkers in Parkinson's disease. Egypt. J. Neurol. Psychiatry Neurosurg. 56:22. doi: 10.1186/s41983-020-0159-7

[B202] Shi M. Kovac A. Korff A. Cook T. J. Ginghina C. Bullock K. M. . (2016). CNS tau efflux via exosomes is likely increased in Parkinson's disease but not in Alzheimer's disease. Alzheimers Dement. 12, 1125–1131. doi: 10.1016/j.jalz.2016.04.003 27234211 PMC5107127

[B203] Shi M. Liu C. Cook T. J. Bullock K. M. Zhao Y. Ginghina C. . (2014). Plasma exosomal alpha-synuclein is likely CNS-derived and increased in Parkinson's disease. Acta Neuropathol. 128, 639–650. doi: 10.1007/s00401-014-1314-y 24997849 PMC4201967

[B204] Si X. Tian J. Chen Y. Yan Y. Pu J. Zhang B. (2019). Central nervous system-derived exosomal alpha-synuclein in serum may be a biomarker in Parkinson's disease. Neuroscience 413, 308–316. doi: 10.1016/j.neuroscience.2019.05.015 31102760

[B205] Siderowf A. Concha-Marambio L. Lafontant D. E. Farris C. M. Ma Y. Urenia P. A. . (2023). Parkinson's progression markers initiative. assessment of heterogeneity among participants in the Parkinson's progression markers initiative cohort using α-synuclein seed amplification: a cross-sectional study. Lancet. Neurol. 22, 407–417. doi: 10.1016/S1474-4422(23)00109-6 37059509 PMC10627170

[B206] Siderowf A. Xie S. X. Hurtig H. Weintraub D. Duda J. E. Chen-Plotkin A. . (2010). CSF amyloid beta 1-42 predicts cognitive decline in Parkinson disease. Neurology 75, 1055–1061. doi: 10.1212/WNL.0b013e3181f39a78 20720189 PMC2942062

[B207] Singer W. Schmeichel A. M. Shahnawaz M. Schmelzer J. D. Boeve B. F. Parisi J. E. . (2020). Alpha-synuclein oligomers and neurofilament light chain in spinal fluid differentiate multiple system atrophy from lewy body synucleinopathies. Ann. Neurol. 88, 503–512. doi: 10.1002/ana.25824 32557811 PMC7719613

[B208] Sproviero D. S. Gagliardi S. Zucca Borsani, G. Lattuada D. (2021). Different miRNA profiles in plasma derived small and large extracellular vesicles from patients with neurodegenerative diseases. Int. J. Mol. Sci. 22:08. doi: 10.3390/ijms22052737 33800495 PMC7962970

[B209] Stav A. L. Aarsland D. Johansen K. K. Hessen E. Auning E. Fladby T. (2015). Amyloid-β and α-synuclein cerebrospinal fluid biomarkers and cognition in early Parkinson's disease. Parkinsonism. Relat. Disord. 21,758–764. doi: 10.1016/j.parkreldis.2015.04.027 25971633

[B210] Stewart T. Sossi V. Aasly J. O. Appel-Cresswell S. Martin W. R. W. Stoessl A. J. (2015). Phosphorylated alpha- synuclein in parkinson's disease: correlation depends on disease severity. Acta Neuropathol. Commun. 3:7. doi: 10.1186/s40478-015-0185-3 25637461 PMC4362824

[B211] Stuendl A. Kraus T. Chatterjee M. Greff F. M. Schmid B. Krause S. M. . (2021). alpha-Synuclein in plasma-derived extracellular vesicles is a potential biomarker of Parkinson's disease. Mov. Disord. 36, 2508–2518. doi: 10.1002/mds.28639 34002893

[B212] Stuendl A. Kunadt M. Kruse N. Klucken J. Koch J. C. Giese A. . (2016). Induction of alpha- synuclein aggregate formation by CSF exosomes from patients with Parkinson's disease and dementia with Lewy bodies. Brain 139, 481–494. doi: 10.1093/brain/awv346 26647156 PMC4805087

[B213] Su W. Chen H. B. Li S. H. Zhang X. Y. Wang L. Liu Y. . (2012). Correlational study of the serum levels of the glial fibrillary acidic protein and neurofilament proteins in Parkinson's disease patients. Clin. Neurol. Neurosurg. 114, 372–375. doi: 10.1016/j.clineuro.2011.11.002 22206859

[B214] Sulzer D. Alcalay R. N. Garretti F. Cote L. Kanter E. Greenspan N. S. . (2017). T cells from patients with Parkinson's disease recognize alpha-synuclein peptides. Nature 546, 656–661. doi: 10.1038/nature22815 28636593 PMC5626019

[B215] Swallow D. M. A. Counsell C. E. (2023). The evolution of diagnosis from symptom onset to death in progressive supranuclear palsy (PSP) and corticobasal degeneration (CBD) compared to Parkinson's disease (PD). J. Neurol. 270, 3464–3474. doi: 10.1007/s00415-023-11629-x 36971841 PMC10266988

[B216] Takov K. Yellon D. M. Davidson S. M. (2018). Comparison of small extracellular vesicles isolated from plasma by ultracentrifugation or size-exclusion chromatography: yield, purity and functional potential. J. Extracell. Vesicles 8:1560809. doi: 10.1080/20013078.2018.1560809 30651940 PMC6327926

[B217] Tang Y. Han L. Li S. Zhang X. Wang J. Liu Y. . (2023). Plasma GFAP in Parkinson's disease with cognitive impairment and its potential to predict conversion to dementia. NPJ Parkinsons Dis. 9:23. doi: 10.1038/s41531-023-00447-7 36759508 PMC9911758

[B218] Tian C. Liu G. Gao L. Soltys D. Pan C. Stewart T. . (2019). Erythrocytic alpha-synuclein as a potential biomarker for Parkinson's disease. Transl. Neurodegener. 8:15. doi: 10.1186/s40035-019-0155-y 31123587 PMC6521422

[B219] Tokuda T. Qureshi M. M. Ardah M. T. Varghese S. Shehab S. A. S. Kasai T. . (2010). Detection of elevated levels of α-synuclein oligomers in CSF from patients with Parkinson's disease. Neurology 75, 1766–1772. doi: 10.1212/WNL.0b013e3181fd613b 20962290

[B220] Twohig D. Rodriguez-Vieitez E. Sando S. B. Berge G. Lauridsen C. Oksengård A. R. . (2018). The relevance of cerebrospinal fluid α-synuclein levels to sporadic and familial Alzheimer's disease. Acta Neuropathol. Commun. 6:130. doi: 10.1186/s40478-018-0624-z 30477568 PMC6260771

[B221] Usenko T. S. Nikolaev M. A. Miliukhina I. V. Bezrukova A. I. Senkevich K. A. Gomzyakova N. A. . (2020). Plasma cytokine profile in synucleinophaties with dementia. J. Clin. Neurosci. 78, 323–326. doi: 10.1016/j.jocn.2020.04.058 32336641

[B222] Vallelunga A. Ragusa M. Di Mauro S. Iannitti T. Puzzo L. Giunta M. L. . (2014). Identification of circulating microRNAs for the differential diagnosis of Parkinson's disease and multiple system atrophy. Front. Cell. Neurosci. 8:156. doi: 10.3389/fncel.2014.00156 24959119 PMC4051126

[B223] van Steenoven I. Aarsland D. Weintraub D. Londos E. Blanc F. van der Flier W. M. . (2016). Cerebrospinal fluid alzheimer's disease biomarkers across the spectrum of Lewy body diseases: results from a large multicenter cohort. J. Alzheimers. Dis. 54, 287–295. doi: 10.3233/JAD-160322 27567832 PMC5535729

[B224] van Steenoven I. Majbour N. K. Vaikath N. N. Berendse H. W. van der Flier W. M. van de Berg W. D. J. . (2018). Alpha-Synuclein species as potential cerebrospinal fluid biomarkers for dementia with Lewy bodies. Mov Disord 33, 1724–1733. doi: 10.1002/mds.111 30440090 PMC6519232

[B225] van Wamelen D. J. Taddei R. N. Calvano A. Titova N. Leta V. Shtuchniy I. . (2020). Serum uric acid levels and non-motor symptoms in Parkinson's disease. J. Parkinsons Dis. 10, 1003–1010. doi: 10.3233/JPD-201988 32444561

[B226] Veerman R. E. Teeuwen L. Czarnewski P. Güclüler Akpinar G. Sandberg A. . (2021). Molecular evaluation of five different isolation methods for extracellular vesicles reveals different clinical applicability and subcellular origin. J. Extracell. Vesicles 10:e12128. doi: 10.1002/jev2.12128 34322205 PMC8298890

[B227] Vesely B. Dufek M. Thon V. Brozman M. Királová S. Halászová T. . (2018). Interleukin 6 and complement serum level study in Parkinson's disease. J. Neural. Transm. 125, 875–881 doi: 10.1007/s00702-018-1857-5 29435648

[B228] Vesely B. Koritakova E. Bohnen N. I. Viszlayova D. Kiralova S. Valkovič P. . (2019). The contribution of cerebrovascular risk factors, metabolic and inflammatory changes to cognitive decline in Parkinson's disease: preliminary observations. J. Neural. Transm. 126, 1303–1312. doi: 10.1007/s00702-019-02043-7 31332506 PMC6959128

[B229] Villar-Menéndez I. Porta S. Buira S. P. Pereira-Veiga T. Díaz-Sánchez S. Albasanz J. L. . (2014). Increased striatal adenosine A2A receptor levels is an early event in Parkinson's disease-related pathology and it is potentially regulated by miR-34b. Neurobiol. Dis. 69, 206–214. doi: 10.1016/j.nbd.2014.05.030 24892887

[B230] Vivacqua G. Latorre A. Suppa A. Nardi M. Pietracupa S. Mancinelli R. . (2016). Abnormal salivary total and oligomeric alpha-synuclein in Parkinson's disease. PLoS ONE 11:e0151156. doi: 10.1371/journal.pone.0151156 27011009 PMC4807094

[B231] Vivacqua G. Suppa A. Mancinelli R. Belvisi D. Fabbrini A. Costanzo M. . (2019). Salivary alpha- synuclein in the diagnosis of Parkinson's disease and progressive supranuclear palsy. Parkinsonism Relat. Disord. 63, 143–148. doi: 10.1016/j.parkreldis.2019.02.014 30796010

[B232] Vranová H. P. Hényková E. Kaiserová M. Menšíková K. Vaštík M. Mareš J. . (2014). Tau protein, beta-amyloid1–42 and clusterin CSF levels in the differential diagnosis of Parkinsonian syndrome with dementia. J. Neurol. Sci. 343, 120–124. doi: 10.1016/j.jns.2014.05.052 24928081

[B233] Wang H. Atik A. Stewart T. Ginghina C. Aro P. Kerr K. F. . (2018). Plasma alpha-synuclein and cognitive impairment in the parkinson's associated risk syndrome: a pilot study. Neurobiol. Dis. 116, 53–59. doi: 10.1016/j.nbd.2018.04.015 29705185 PMC6294306

[B234] Wang S. Liu Z. Ye T. Mabrouk O. S. Maltbie T. Aasly J. . (2017). Elevated LRRK2 autophosphorylation in brain-derived and peripheral exosomes in LRRK2 mutation carriers. Acta Neuropathol. Commun. 5:86. doi: 10.1186/s40478-017-0492-y 29166931 PMC5700679

[B235] Wang X. Yu S. Li F. Feng T. (2015). Detection of alpha-synuclein oligomers in red blood cells as a potential biomarker of Parkinson's disease. Neurosci. Lett. 599,115–119. doi: 10.1016/j.neulet.2015.05.030 25998655

[B236] Wang Y. Hu J. Chen X. Wang S. Zhang C. Hu J. . (2022). Real-time quaking-induced conversion assay is accurate for Lewy body diseases: a meta-analysis. Neurol. Sci. 43, 4125–4132. doi: 10.1007/s10072-022-06014-x 35312879

[B237] Wang Y. Shi M. Chung K. A. Zabetian C. P. Leverenz J. B. Berg D. . (2012). Phosphorylated alpha- synuclein in Parkinson's disease. Sci. Transl. Med. 4:121ra20. doi: 10.1126/scitranslmed.3002566 22344688 PMC3302662

[B238] Wang Z. Gililand T. Kim H. J. Garasimenko M. Sajewski K. . (2024). A minimally invasive biomarker for sensitive and accurate diagnosis of Parkinson's disease. Acta Neuropathol. Commun. 12:167. doi: 10.1186/s40478-024-01873-1 39439002 PMC11495072

[B239] Welsh J. A. Goberdhan D. C. I. O'Driscoll L. Buzas E. I. Blenkiron C. Bussolati B. . (2024). Minimal information for studies of extracellular vesicles (MISEV2023): from basic to advanced approaches. J. Extracell. Vesicles 13:e12404. doi: 10.1002/jev2.12404 38326288 PMC10850029

[B240] Wennstrom M. Surova Y. Hall S. Nilsson C. Minthon L. Hansson O . (2015). The inflammatory marker YKL-40 is elevated in cerebrospinal fluid from patients with Alzheimer's but not Parkinson's disease or dementia with Lewy bodies. PLoS ONE 10:e0135458. doi: 10.1371/journal.pone.0135458 26270969 PMC4536228

[B241] White A. J. Wijeyekoon R. S. Scott K. M. Gunawardana N. P. Hayat S. Solim I. H. . (2018). The peripheral inflammatory response to alpha-synuclein and endotoxin in Parkinson's disease. Front. Neurol. 9:946. doi: 10.3389/fneur.2018.00946 30524354 PMC6256248

[B242] Williams S. M. Schulz P. Sierks M. R. (2016). Oligomeric α-synuclein and β-amyloid variants as potential biomarkers for Parkinson's and Alzheimer's diseases. Eur. J. Neurosci. 43, 3–16. doi: 10.1111/ejn.13056 26332448 PMC4718789

[B243] Xylaki M. Chopra A. Weber S. Bartl M. Outeiro T. F. Mollenhauer B. (2023). Extracellular vesicles for the diagnosis of parkinson's disease: systematic review and meta-analysis. Mov. Disord. 38, 1585–1597 doi: 10.1002/mds.29497 37449706

[B244] Yan S. Jiang C. Janzen A. Barber T. R. Seger A. Sommerauer M. . (2024). Neuronally derived extracellular vesicle alpha-synuclein as a serum biomarker for individuals at risk of developing Parkinson disease. JAMA Neurol. 81, 59–68. doi: 10.1001/jamaneurol.2023.4398 38048087 PMC10696516

[B245] Yanamandra K. Gruden M. A. Casaite V. Meskys R. Forsgren L. Morozova-Roche L. A. (2011). alpha-Synuclein reactive antibodies as diagnostic biomarkers in blood sera of Parkinson's disease patients. PLoS ONE 6:e18513. doi: 10.1371/journal.pone.0018513 21541339 PMC3081826

[B246] Yilmaz R. Strafella A. P. Bernard A. Schulte C. van den Heuvel L. . (2018). Serum inflammatory profile for the discrimination of clinical subtypes in Parkinson's disease. Front. Neurol. 9:1123. doi: 10.3389/fneur.2018.01123 30622507 PMC6308160

[B247] Youssef P. Hughes L. Kim W. S. Glenda M. Halliday Simon J. G. . (2023). Evaluation of plasma levels of NFL, GFAP, UCHL1 and tau as Parkinson's disease biomarkers using multiplex single molecule counting. Sci. Rep. 13“5217. doi: 10.1038/s41598-023-32480-0 36997567 PMC10063670

[B248] Youssef P. Kim W. S. Halliday G. M. Lewis S. J. G. Dzamko N. (2021). Comparison of different platform immunoassays for the measurement of plasma alpha-synuclein in Parkinson's disease patients. J. Parkinsons. Dis. 11:1761. doi: 10.3233/JPD-212694 34151860 PMC8609717

[B249] Zanganeh S. Arias G. Cone A. Yuan R. Dittmer D. P. (2026). Protocol for large-scale, high-yield, high-purity extracellular vesicle purification from human plasma. STAR Protoc. 7:104428. doi: 10.1016/j.xpro.2026.104428 41818236 PMC12994032

[B250] Zhao Y. Yang G. F. (2021). Potential of extracellular vesicles in the Parkinson's disease-pathological mediators and biomarkers. Neurochem. Int. 144:104974. doi: 10.1016/j.neuint.2021.104974 33485881

[B251] Zhao Z. H. Chen Z. T. Zhou R. L. Zhang X. Ye Q. Y. Wang Y. Z. (2019). Increased DJ-1 and alpha-synuclein in plasma neural-derived exosomes as potential markers for Parkinson's disease. Front. Aging Neurosci. 10:438. doi: 10.3389/fnagi.2018.00438 30692923 PMC6339871

[B252] Zheng H. Xie Z. Zhang X. Mao J. Wang M. Wei S. . (2021). Investigation of alpha-synuclein species in plasma exosomes and the oligomeric and phosphorylated alpha-synuclein as potential peripheral biomarker of Parkinson's disease. Neuroscience 469, 79–90. doi: 10.1016/j.neuroscience.2021.06.033 34186110

[B253] Zhou B. Wen M. Yu W. F. Zhang C. L. Jiao L. (2015). The diagnostic and differential diagnosis utility of cerebrospinal fluid alpha -synuclein levels in Parkinson's disease: a meta-analysis. Parkinson Dis. 2015:567386. doi: 10.1155/2015/567386 26336612 PMC4532865

[B254] Zimmerman M. Brockman K. (2022). Blood and cerebrospinal fluid biomarkers of inflammation in Parkinson's disease. J. Parkinson Dis. 12, S183–S200. doi: 10.3233/JPD-223277 35661021 PMC9535573

[B255] Zubelzu M. Morera-Herreras T. Irastorza G. Gómez-Esteban J. C. Murueta-Goyena A. (2022). Plasma and serum alpha-synuclein as a biomarker in parkinson's disease: a meta-analysis. Parkinsonism Relat. Disord. 99, 107–115. doi: 10.1016/j.parkreldis.2022.06.001 35717321

